# Machine Learning Uses Chemo-Transcriptomic Profiles to Stratify Antimalarial Compounds With Similar Mode of Action

**DOI:** 10.3389/fcimb.2021.688256

**Published:** 2021-06-29

**Authors:** Ashleigh van Heerden, Roelof van Wyk, Lyn-Marie Birkholtz

**Affiliations:** ^1^ Department of Biochemistry, Genetics and Microbiology, University of Pretoria, Hatfield, South Africa; ^2^ University of Pretoria Institute for Sustainable Malaria Control, University of Pretoria, Hatfield, South Africa

**Keywords:** machine learning, mode of action, gene expression profile, biomarker, multinominal logistic regression, chemo-transcriptomic fingerprint

## Abstract

The rapid development of antimalarial resistance motivates the continued search for novel compounds with a mode of action (MoA) different to current antimalarials. Phenotypic screening has delivered thousands of promising hit compounds without prior knowledge of the compounds’ exact target or MoA. Whilst the latter is not initially required to progress a compound in a medicinal chemistry program, identifying the MoA early can accelerate hit prioritization, hit-to-lead optimization and preclinical combination studies in malaria research. The effects of drug treatment on a cell can be observed on systems level in changes in the transcriptome, proteome and metabolome. Machine learning (ML) algorithms are powerful tools able to deconvolute such complex chemically-induced transcriptional signatures to identify pathways on which a compound act and in this manner provide an indication of the MoA of a compound. In this study, we assessed different ML approaches for their ability to stratify antimalarial compounds based on varied chemically-induced transcriptional responses. We developed a rational gene selection approach that could identify predictive features for MoA to train and generate ML models. The best performing model could stratify compounds with similar MoA with a classification accuracy of 76.6 ± 6.4%. Moreover, only a limited set of 50 biomarkers was required to stratify compounds with similar MoA and define chemo-transcriptomic fingerprints for each compound. These fingerprints were unique for each compound and compounds with similar targets/MoA clustered together. The ML model was specific and sensitive enough to group new compounds into MoAs associated with their predicted target and was robust enough to be extended to also generate chemo-transcriptomic fingerprints for additional life cycle stages like immature gametocytes. This work therefore contributes a new strategy to rapidly, specifically and sensitively indicate the MoA of compounds based on chemo-transcriptomic fingerprints and holds promise to accelerate antimalarial drug discovery programs.

## Introduction

Tremendous progress has been made to decrease clinical incidences of malaria by 40% in Africa but the global increase in malaria cases in 2017/8 (World Health Organization, 2019), together with continued antimalarial resistance development, highlights the fragile nature of malaria elimination strategies. Disease pathogenesis is caused by *Plasmodium falciparum* as the most lethal form, and occurs when erythrocytes are infected as host cells for the asexual replication cycle of the parasite, a repetitive process occurring every ~48 h. Transmission to mosquito vectors is ensured by the stochastic development of sexual gametocyte forms from a minor proportion of asexual parasites (Josling et al., 2018), with mature gametocytes able to be taken up by a feeding female *Anopheles* mosquito. Sexual replication of the parasite subsequently occurs in the mosquito to ultimately form sporozoites that are able to be transmitted back to humans. Gametocytogenesis in *P. falciparum* is a uniquely extended process of 10-14 days and relies on development from immature early-stage (morphologically distinguished stage I-III) to late-stage (stage IV/V) gametocytes, prior to maturation to the transmissible stage V gametocytes.

New chemical compounds are required to support malaria elimination efforts and these compounds should have the ability to target multiple stages of the malaria parasite’s development (Birkholtz et al., 2016; Burrows et al., 2017). Additionally, such compounds should have a novel mode of action (MoA) and will be used in combination with each other to lower the rate of resistance emergence (Verlinden et al., 2016). Phenotypic whole-cell screening has successfully delivered thousands of hit compounds [validated hits (Quancard et al., 2021)] with nanomolar whole-cell activity against multiple life cycle stages of *P. falciparum* (Plouffe et al., 2008; Gamo et al., 2010; Delves, 2012; Miguel-Blanco et al., 2017; Delves et al., 2018; Delves et al., 2019; Abraham et al., 2020; Reader et al., 2021). However, this process is not guided by any knowledge on a compounds’ MoA or target. The latter is typically only determined during hit-2-lead (H2L) or lead optimization (LO) phases of the drug discovery process (Yang et al., 2021), and could decrease the timely progression of compounds through the drug discovery pipeline. H2L optimization requires iterative modification of hits to establish structure-activity relationships. This is used to then guide chemical modifications that increase a compound’s potency whilst addressing toxicity and safety issues. Whilst hit validation processes are standardized to streamline the phenotypic screening process (Quancard et al., 2021), H2L optimization, however, is fraught with the possibility that changes in potency as a result of chemical modifications can be due to a change in the derivative’s MoA and binding of the compound to a different target, presenting with ‘off-target’ or toxic effects. Not only this, but compounds with undesired MoAs may only be discovered during the LO phase when the cost invested in such leads is already incurred. The challenge is therefore to devise a tool that can be used in these initial stages of a drug discovery program to quickly, efficiently and economically stratify a compound, and its derivatives, based on their potential MoA.

Several approaches are currently used to describe an antimalarial compound’s MoA and includes *in vitro* evolution and whole genome sequencing to identify chemically validated targets (Yang et al., 2021). Beyond direct target identification, ‘-Omics’ approaches have been useful to describe the MoA ‘fingerprint’ of a compound as a descriptor of a compound’s induced phenotype on e.g. the transcriptome or metabolome (Hu et al., 2009; Allman et al., 2016). This has identified targeted pathways for several frontrunner compounds and (pre-)clinical antimalarial candidates (Allman et al., 2016). However, only a handful of metabolites can be quantitatively detected and stage comparison may be difficult as some stages of the parasite are more metabolically active than others (Gulati et al., 2015). Some compounds whose MoA is unrelated to metabolism may be more difficult to ascertain or detect (Creek et al., 2016; Tulloch et al., 2018). This, together with the time and resource-intensive nature precludes the at scale use of metabolomics to guide the profiling of compounds early on in the drug discovery pipeline.

Genome-wide expression profiling of chemically-induced changes in transcriptomes have successfully elucidated the MoA and even identified possible targets of antineoplastic agents (Iwata et al., 2017). Indeed, the connectivity map (CMap) database contains gene expression profiles of five cancer cell lines perturbed by over 5000 compounds (Sirci et al., 2016). These profiles are all integrated through gene expression network analyses to indicate either those processes induced to overcome the drug effect and retain homeostatic control, or alternatively indicate the direct effect of a compound on a cellular pathway (Isik et al., 2015; Woo et al., 2015). Although *P. falciparum* relies on tight control of gene expression to regulate various processes during its complex life cycle (Bozdech et al., 2003; van Biljon et al., 2019), reproducible, specific and unique chemically-induced changes in gene expression patterns can be elicited. Asexual blood stage parasites treated with compounds with different MoAs show distinct and divergent transcriptomic responses (Hu et al., 2009; Siwo et al., 2015; van der Watt et al., 2018). This has also been observed for immature gametocytes (Ngwa et al., 2017; Ngwa et al., 2019; Reader et al., 2021), which implies that chemically-induced transcriptome responses can be detected for various life cycle stages of the parasite. The parasite’s transcriptome can therefore be mined to stratify compounds to specific MoAs. However, this requires extensive deconvolution of the complex transcriptome profiles obtained as a result of the perturbation and may again not be feasible to perform at scale for multiple compounds during e.g. H2L phases.

Machine learning (ML) algorithms provide powerful, unbiased approaches to deconvolute complex transcriptional drug signatures in order to either identify pathways on which a compound act or describe the global phenotypic effect of inhibition of a particular drug target (Tan et al., 2018). One benefit that these algorithms have over gene interaction network analysis methods, is that there is no prerequisite for defining in detail the structure of interaction between genes (Melas et al., 2013). ML has already successfully delineated the MoA of various compounds/drugs in human cells in a cell-specific manner and at scale (Zhang et al., 2016; Iwata et al., 2017), and was even able to predict drug targets in cancer studies (Hizukuri et al., 2015; Pabon et al., 2018; Sawada et al., 2018; Xie et al., 2018). However, aside from some reports where large datasets of phenotypic screening data were used to predictively identify antiplasmodial hit compounds using transfer and deep learning (Keshavarzi Arshadi et al., 2019), the use of ML to define chemically-induced transcriptome signatures for MoA classification has not been explored in depth for *P. falciparum*.

We aimed to develop a ML model that can stratify potential antimalarial compounds with similar MoA based on their chemically-induced transcriptional responses on asexual stages of *P. falciparum* parasites. We show that only a limited number of differentially expressed genes (DEGs), that are unique for a MoA and pervasive throughout a compound’s treatment, was needed as predictive features to train a robust MoA stratification model. These biomarkers could be used to generate chemo-genomic fingerprints for compounds associated with similar MoA. The ML model and biomarkers could be used at scale to enable medium through-put MoA elucidation and guide preclinical decisions to fast-track H2L optimization in drug discovery.

## Materials and Methods

### Data Acquisition, Quality Control Filtering, and Pre-Processing of GEP Datasets

**Figure 1:**
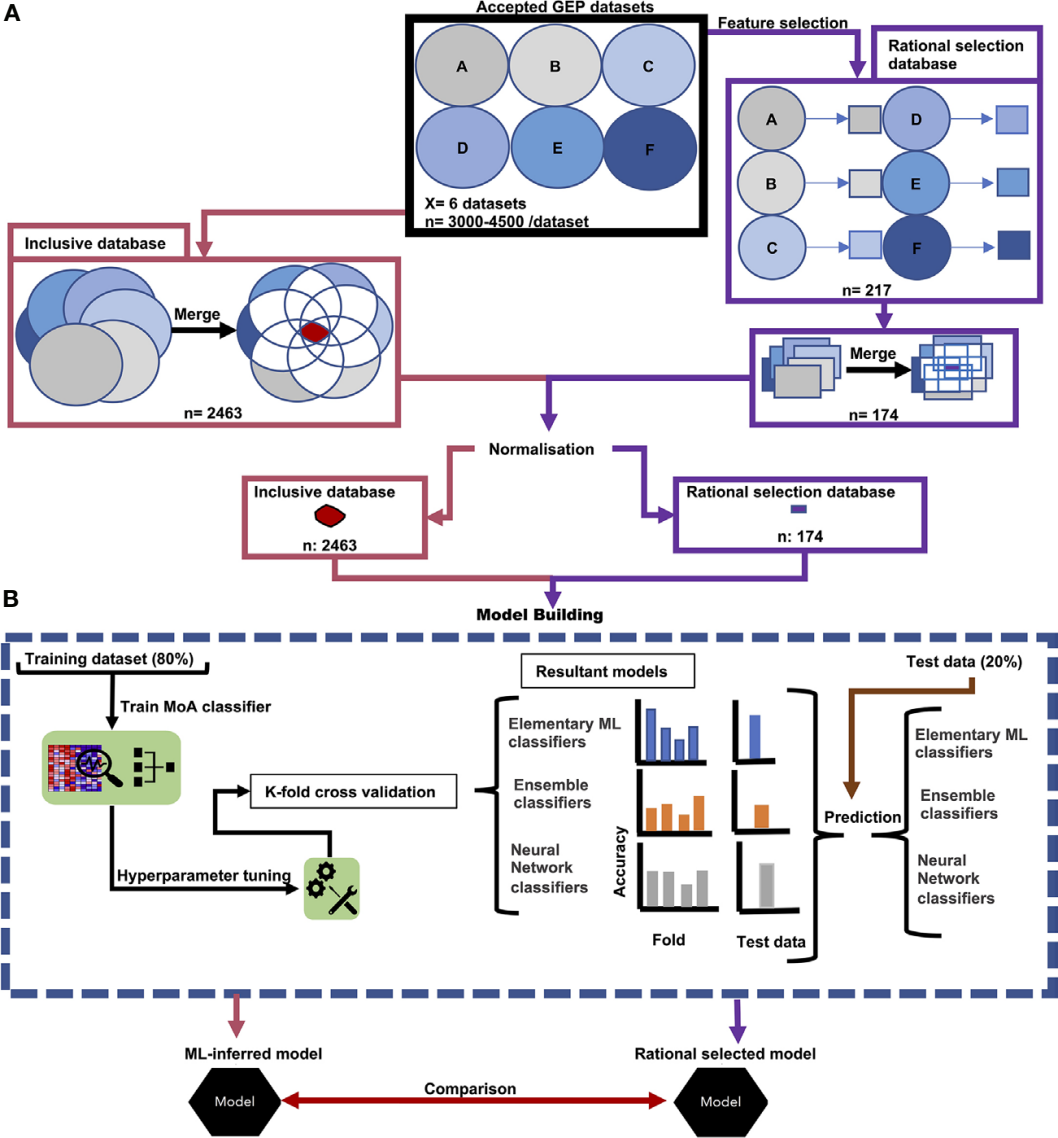
Workflow of data acquisition and model building. **(A)** Accepted GEPs of treated *P. falciparum* formed our inclusive database (pink) and underwent feature-selection (purple). In both instances the GEPs were merged and underwent normalization. The resultant transcripts from the (pink) accepted GEPs produced our inclusive database. Similarly, the consequential transcripts from our feature selection produced our rational selection database (purple). **(B)** Both transcripts in the inclusive database and in the rational selection database were used to separately build models. Treatment time points were randomly split at an 80/20 ratio, whereby 80% was used as a training set for model tuning, training and K-fold cross validation. The resultant 20% of treatment time points were used as a test set to evaluate model performance on untrained data. Prediction accuracy of treatment MoA from both the K-fold cross validation and prediction on the test set was used to determine the best classification algorithm for antiplasmodial MoA. From this classification algorithm, the resultant models trained on either the inclusive database or rational selection database were compared and assessed which selection approach was the best in identifying the minimum number of transcripts for robust MoA prediction using a sliding gene-scale approach.

An in-house database was generated consisting of gene expression profiles (GEPs) of *P. falciparum* parasites treated with different compounds (see **Supplementary Table S1**). Each GEP dataset was assessed individually for quality and inclusion based on the filtering criteria in **Supplementary Table S2**. Inclusion criteria included the presence of acceptable and comparable controls, >65% coverage of the *P. falciparum* transcriptome, treatment with known MoA, ≥2 time points available, IC_50_ or higher concentrations and an acceptable parasite strain used. Accepted datasets with accession codes GSE19468, GSE13578, GSE1 00692, GSE39485, GSE33869, GSE19468 and GSE25642 were downloaded from Gene Expression Omnibus (GEO) before being merged to form a GEP database (**Figure 1**). To negate platform differences, between-array normalization was required to enable comparison between datasets. This was also an essential pre-processing step before the data could be used to build a model using machine learning algorithms, as some algorithms require normalized data. Different microarray between-array normalization strategies were assessed for their ability to allow comparisons between different GEPs using the limma package (version 3.46.0) in R (Ritchie et al., 2015). Ultimately, cyclic loess normalization performed the best (**Supplementary Figure S1**) and was applied to all the data in the database.

### Feature Selection

To identify features i.e., transcripts that may be useful for MoA stratification and that would be used to build the ML models, a rational transcript selection criterion was developed (**Supplementary Figure S2**). Transcripts that fell within the upper or lower 5^th^ percentile of expression in the GEP during any time point in a compound treatment were defined as differentially expressed (DE). These selection criteria for the rational selection included: 1) removal of non-informative transcripts that are not significantly DE: 2) removal of DEGs that display discontinuous DE profiles throughout treatment with a compound and vary across time points. Such DEGs may not function as predictive features and were removed to reduce noise and variability in the model; 3) only genes that were continuously DE across all time points of a treatment was selected; 4) to prevent inclusion of DEGs that are due to general drug stress or other factors, those shared between compound treatments were excluded.

In addition to the above rational selection, an objective transcript selection approach was also applied, whereby ML-inferred top features were identified through variable importance by the best antiplasmodial MoA stratification ML algorithm. The database model generated by the algorithm after tuning and training on the transcripts for all 2463 genes, was used to rank transcripts according to their variable importance i.e., importance a transcript has in aiding a model in MoA stratification.

### Supervised ML Models

Three different algorithm categories were investigated. Firstly, elementary ML algorithms for multiclass classification problems such as support vector classification (SVC) and multinomial logistic regression (MLR) that use statistics were investigated. Secondly, ensemble classifiers algorithms like random forest (RF) and gradient boosting machines (GBM) were also examined as they combine multiple shallow learner models produced during training to create a more optimal model. Lastly, deep learning algorithms that create artificial neural networks (ANN) was also explored due to how these ANN uses multiple layers to extract higher-level features from data.

These SVC, MLR, RF, GBM and ANN algorithms were evaluated for their ability to create a stable and robust antiplasmodial MoA stratification model from transcriptomic data. Transcripts were used as training features and their respective expression values within a specific treatment time point were used as the input data points. Compound treatments were labelled according to their respective MoA and the collection of treatment time points were randomly split into training and testing sets at an 80:20 ratio. Model hyperparameters define a model’s architecture and affect model performance, thus to ensure models were optimized and had the optimal architecture to address the MoA classification problem, hyperparameters (e.g. number of trees in the case of RF and GBM) were fine-tuned individually for each algorithm using the training set (**Supplementary Table S4**) (Schratz et al., 2019).

To build a MLR model for multiclassification, the h2o (version 3.32.0.1) R package was used by employing the h2o.glm() function, which is abbreviated for a generalized linear model and can be used for both binary and multiclassification problems (Cawley et al., 2007; LeDell et al., 2019), and for which hyperparameter tuning could be excluded (Moreira et al., 2018).

Multiclass SVC models were built with the e1071 (version 1.7-6) R package to train and fine-tune the hyperparameters required (Meyer et al., 2019), and optimal hyperparameters were identified based on classification error. Different kernels were also investigated as they are mathematical functions often used within SVCs to transform input data into a desirable form whereby patterns can be more readily discovered. During hyperparameter tuning, no assumptions were made regarding the data space and hence various kernels (sigmoid, polynomial, linear and radial) were assessed for their MoA stratification performance.

Variations of RF models for multiclassification were built using either the randomForest (version 4.6‑14), or the h2o R package (Liaw and Wiener, 2002; LeDell et al., 2019). Hyperparameter tuning was implemented using the e1071 R package for randomForest models and an internal grid search function was used for hyperparameter tuning for the h2o models (Meyer et al., 2019). Classification error was used to identify the optimal hyperparameters, however, for the mtries hyperparameter the out-of-bag error was used to identify the optimal hyperparameter value. With the h2o package hyperparameters were selected with the lowest Logloss value (Brownlee, 2016).

Similarly to RF, variations of GBM models were built either using either the xgboost (version 1.3.2.1) R package or the h2o R package (Chen et al., 2019; LeDell et al., 2019). Hyperparameter tuning was implemented using the caret R package for xgboost models and internal grid search function was used for hyperparameter tuning for the h2o models (Kuhn et al., 2019; LeDell et al., 2019). The caret package used classification accuracy as a performance measure in selecting the optimal hyperparameters, whereas the h2o package used Logloss to select the optimal hyperparameters.

The h2o R package with the h2o.deeplearning() function was used to build a ANN capable of multiclassification and an internal grid search was done to find the optimal model hyperparameters using Logloss as a performance measure (LeDell et al., 2019). Due to computational cost and efficiency, not all the neural network hyperparameters and/or large ranges could be investigated (see **Supplementary Table S4**).

### Assessing Different ML Algorithms to Stratify Antiplasmodial Compounds With Similar MoA

A 10-fold cross-validation was performed during model training to assess model stability and to obtain a more accurate metric to assess model accuracy. The test set was used as an external validation of models on untrained data. Both were used to assess overfitting of the models. The caret package was used to perform a 10-fold cross-validation on the RF and SVC models made using the randomForest and e1071 R packages, respectively (Liaw and Wiener, 2002; Kuhn et al., 2019; Meyer et al., 2019). In instances where the h2o R package was used to make models (such as the ANN, MLR, RF and GBM), the 10-fold cross-validation was done simultaneously without the requirement of another RStudio package (LeDell et al., 2019).

For downstream selection analysis, each algorithm was used to construct two ML models: 1) one model trained on all 2463 transcripts within the inclusive database, which excluded all filtering or selection and includes all the available transcripts in the database; and 2) the other model trained on only the 174 transcripts identified from the rational gene selection criterion (**Supplementary Figure S2**) i.e., the rational selection database. The best algorithm was selected based on results from the 10-fold cross-validation, model stability and classification accuracy on test data of models trained on the 2463-transcript inclusive database and those trained on 174-transcript rational selection database.

### Comparison of the Rational Selection and ML-Inferred Selection Strategies

Feature selection is an essential part of ML as it helps reduce the dimensionality of high dimensionality datasets such as GEP data and also improves predictive accuracy by focusing on data that is relevant for the model (Motoda and Liu, 2002).

We evaluated if the transcripts identified from our rational selection approach (defined as genes selected based on criterion defined in *Feature Selection* as per **Supplementary Figure S2**) compared to transcripts objectively identified in an unsupervised fashion by the ML algorithm with good predictive value. To prevent unintended bias in selection, the 2463-transcript inclusive database model was used by the best ML algorithm to rank transcripts according to variable importance for MoA stratification when determining compound MoA. Variable importance is an indication of how much the model relies on that variable to make accurate predictions. From these top ML-inferred transcripts, a model was built using the same number of transcripts (174) that was present in the 174-transcript rational selection database model to allow proper computational comparison between the two models. Based on results from the 10-fold cross validation, model stability and classification accuracy on test data the best gene selection approach (ML-inferred *vs*. rational selection) was identified.

To further ensure that the best selection method was identified, a sliding gene-scale approach was applied on both the ML-inferred transcripts and the rationally selected transcripts. Transcripts were ranked according to variable importance in MoA stratification. From this ‘minimodels’ were made for both selection approaches with each sequential model containing fewer transcripts to train on than the previous model. The minimodel that performed the best in their classification accuracy, model stability and test set (untrained data) using the least number of transcripts determined which approach was more suitable for gene selection. The theory is that as the number of transcripts decreases, transcripts that are noisy or redundant will become more apparent in affecting the model’s performance by lowering the accuracy of the model, whereas the opposite is true for good predictive transcripts. From this also the minimum number of transcripts for robust MoA stratification was determined.

To highlight any loss in model performance and overfitting for minimodels due to the reduced number of transcripts used for training, leave-one-out cross validation (LOOCV) was implemented, and the root mean squared error (RMSE), average log-loss and LOOCV correlation coefficient (Q^2^LOO) calculated for each minimodel. Minimodels which indicated low values for RMSE and log-loss as well as a decreased difference between their R^2^ and Q^2^LOO were considered as models with good fit to training data.

### Validation of the ML Model on New Compounds and Chemo-Transcriptomic Fingerprinting

To determine whether unique expression patterns could be associated with the biomarker transcripts that define each compound treatment (**Table 1**), the normalized log_2_ fold change profiles for each of the 50 biomarker transcripts was extracted from the original datasets (**Supplementary Table S3**) for all the treatments used to generate the model (**Table 1**). This was then used to construct an expression profile heatmap using the ggplots2 package (version 3.3.3) (Wickham, 2016) in R. Only one timepoint, where the strongest perturbation within the 50 biomarkers were evident, was included for each compound treatment. Gene transcripts were grouped according to the compound MoA they were identified from (**Table 1**).

**Table 1:** The top 50 biomarkers identified from the ML modeling.

Treatment	Mode of action/target protein	PlasmoDB Gene ID	Gene product description
ML-7 & W7 (Hu et al., 2009)	Ca^2+^/calmodulin-dependent protein kinase inhibitor	*pf3D7_0108700*	Secreted ookinete protein
		*pf3D7_0203700*	protein MAK16
		*pf3D7_0206700*	Adenylosuccinate lyase
		*pf3D7_0508700*	Pre-mRNA-processing ATP-dependent RNA helicase PRP5
		*pf3D7_0618100*	Conserved *Plasmodium* protein, unknown function
		*pf3D7_1308500*	Conserved *Plasmodium* protein, unknown function
TSA (Hu et al., 2009) SAHA, TSA & ASA-9 (Andrews et al., 2012)	Histone deacetylase	*pf3D7_0322100*	mRNA-capping enzyme subunit beta
		*pf3D7_0614300*	Major facilitator superfamily-related transporter
		*pf3D7_1015500*	Nucleotidyltransferase
		*pf3D7_1039000*	Serine/threonine protein kinase, FIKK family
		*pf3D7_1112700*	Conserved *Plasmodium* protein, unknown function
		*pf3D7_1323800*	Vacuolar protein sorting-associated protein 52
		*pf3D7_1438000*	Eukaryotic translation initiation factor eIF2A
Febrifugine (Hu et al., 2009)	Prolyl-tRNA synthetase	*pf3D7_0623900*	Ribonuclease H2 subunit A
		*pf3D7_1030600*	tRNA N6-adenosine threonylcarbamoyltransferase
		*pf3D7_1467400*	50S ribosomal protein L22, apicoplast
Staurosporine A (Hu et al., 2009)	Serine/threonine kinases	*pf3D7_0206100*	Cysteine desulfuration protein SufE
		*pf3D7_0619800*	Conserved *Plasmodium* membrane protein, unknown function
		*pf3D7_0806600*	Kinesin-like protein
		*pf3D7_1220400*	Debranching enzyme-associated ribonuclease
		*pf3D7_1317100*	DNA replication licensing factor MCM4
Artemisinin (Hu et al., 2009)	Free radicals formation and protein & heme alkylation	*pf3D7_1325400*	Conserved *Plasmodium* protein, unknown function
		*pf3D7_1475100*	Conserved *Plasmodium* protein, unknown function
DFMO (van Brummelen et al., 2008)	Ornithine decarboxylase	*pf3D7_0503400*	Actin-depolymerizing factor 1
		*pf3D7_0509100*	Structural maintenance of chromosomes protein 4
		*pf3D7_1019800*	tRNA methyltransferase
		*pf3D7_1242700*	40S ribosomal protein S17
		*pf3D7_1425800*	Conserved *Plasmodium* protein, unknown function
		*pf3D7_1440500*	Allantoicase
Cyclosporine A (Hu et al., 2009)	Binds sphingomyelin	*pf3D7_0317300*	Conserved *Plasmodium* protein, unknown function
		*pf3D7_1013500*	Phosphoinositide-specific phospholipase C
		*pf3D7_1127900*	Conserved *Plasmodium* protein, unknown function
		*pf3D7_1352000*	GTP-binding protein
		*pf3D7_1474500*	Splicing factor 3A subunit 1
Chloroquine & Quinine (Hu et al., 2009)	Heme metabolism	*pf3D7_0612600*	Cytoplasmic tRNA 2-thiolation protein 1
		*pf3D7_0704500*	Serine/threonine protein kinase
		*pf3D7_1324000*	Conserved *Plasmodium* protein, unknown function
		*pf3D7_0604100*	AP2 domain transcription factor
		*pf3D7_1322200*	Conserved *Plasmodium* protein, unknown function
		*pf3D7_1427000*	Conserved *Plasmodium* protein, unknown function
PMSF (Hu et al., 2009)	Serine protease	*pf3D7_0511800*	Inositol-3-phosphate synthase
		*pf3D7_0717800*	Conserved *Plasmodium* protein, unknown function
		*pf3D7_0823800*	DnaJ protein
		*pf3D7_1115400*	Cysteine proteinase falcipain 3
		*pf3D7_1458900*	Golgi apparatus membrane protein TVP23
Ionomycin (Cheemadan et al., 2014)	Ca2+-binding ionophore	*pf3D7_1038400*	Gametocyte-specific protein
MMV’048 & UCT’943 (van der Watt et al., 2018)	Phosphatidylinositol 4-kinase (PI4K)	*pf3D7_0213000*	Conserved protein, unknown function
		*pf3D7_0301800*	*Plasmodium* exported protein, unknown function
		*pf3D7_1340900*	Sodium-dependent phosphate transporter
		*pf3D7_1404400*	Ribosomal protein L16, mitochondrial

To further define the grouping within this dataset and reduce the dimensionality of the expression profiles of the 50 transcripts, self-organizing maps (SOMs) were used to cluster and summarize the expression patterns using the supraHex (version 1.28.1) R package (Fang and Gough, 2014). The normalized log2 fold change profiles of these biomarker transcripts were extracted again for all 50 transcripts across all 12 the treatments included in the model building and used to train SOMs. SOMs were subsequently visualized as two-dimensional suprahexagonal chemo-transcriptomic fingerprints, with each hexagon defining a cluster of genes with the same or similar expression patterns. Within the suprahexagonal fingerprints, the most influential biomarkers are allocated to hexagons at the edge, whereas genes with random or no change in expression were allocated to hexagons in the center. The geographical location of the hexagons depicts the similarity to other hexagons. Input data (distribution, distance, identity and relationship for each biomarker transcript) is retained between fingerprints. Additionally to SOMs, the expression profiles of all 50 transcripts across all 12 the treatments were hierarchically clustered using Ward linkage on Euclidian distance of expression profiles and then visualized with dendextend (version 1.14.0) R package and the SOMs fingerprints for the corresponding treatments that clustered together were compared to one another (Murtagh and Legendre, 2014).

Lastly, to interrogate the sensitivity and multifaceted use of the biomarker transcripts, they were further evaluated in their usefulness to classify the MoA of new compounds, without the use of our trained ML model. Two compounds that are closely related to the PI4K kinase inhibitors MMV’048 and UCT’943 were included: MMV666810 and MMV675850 (van der Watt et al., 2018) with transcriptome data obtained from GEO with accession code GSE167068 (van Biljon et al. submitted to this special issue). These compounds were used to treat immature gametocytes, and this allowed an additional dimensionality in our analysis to include stage-specific variability between asexual parasites and immature gametocytes. Additionally, we included a dataset where immature gametocytes were treated with trichostatin A (TSA) (GSE99223) (Ngwa et al., 2017). Correlation plots were generated with the ‘corrplot’ package (version 0.84) in R (Simko, 2017) to visualize similarity in gene expression profiles between different treatments using the 50 biomarker transcripts. Additionally, chemo-transcriptomic fingerprints were generated using SOM for each of the new compounds treated on early gametocytes and where then compared to the chemo-transcriptomic fingerprints of those with similar MoA treated on asexual parasites.

## Results

### Database Generation and Model Building

Two parallel approaches were used to evaluate and compare the performance of different ML algorithms to build MoA stratification models. This was based either on an objective selection approach (which relied on model building and ML-inferred transcript selection without imposing additional filtering and selection criteria, called henceforth the ‘inclusive database’), or to a rational selection approach (where transcripts were specifically and rationally selected based on a set of criteria imposed (described in *Feature Selection* and **Supplementary Figure S2**, called the ‘rational selection database’). In both instances, a smaller database was generated from the complete input dataset (**Figure 1A**). Both databases was subsequently used for ML model building. In each instance, the respective database was randomly fractionated into a training:test set (80:20), and different ML algorithms applied to determine their ability to build an accurate model for MoA stratification. Finally, the best model from the inclusive database was compared to the best model from the rational selection database (**Figure 1B**) based on accuracy scores from 10-fold cross validation, variability indicators and performance on the test set data (the remaining 20%).

In totality, six datasets (each with 3500-4000 transcript profiles) were included to generate our database (**Figure 1A**). These datasets span treatment of asexual parasites with 20 compounds with a variety of MoAs (**Supplementary Table S3**), ranging from cell signaling [Ca^2+^/calmodulin protein kinase (CDPK) inhibitors and serine/threonine kinase inhibitors], to heme metabolism (heme polymerase enzyme inhibitors) and transcription [histone deacetylases (HDAC) inhibitors]. Most of the datasets included had a high transcript coverage at ≥ 65% and treatments in these datasets had 2-5 time points to allow for selection present over a broad timeframe.

The inclusive database was generated by including transcripts that were shared between the six datasets to allow for comparison of GEP under different treatments. Merging of these datasets resulted in a reduction of transcripts from the 3000-4500 per dataset to 2463 transcripts in total in the final inclusive database, after normalization (**Supplementary Figure S1**). This database spans a total of 103 time points for 20 compounds and could be used to explore different ML algorithms for their efficacy and relevancy for subsequent use in MoA stratification. The 2463-transcript inclusive database was then used for building the predictive inclusive models (**Figure 1B**).

In parallel, the six datasets underwent further individual feature selection to extract the minimum informative transcripts to be included in the rational selection database and thereby eliminate non-informative transcripts to improve model performance (**Supplementary Figure S2**). DEGs were identified and extracted that continuously fell within the upper/lower 5^th^ percentile of expression throughout a specific treatment and did not show this behavior towards other treatments with different MoA. This resulted in a stringent reduction to an exclusive 217 unique DEGs that was further reduced to only 174 (**Supplementary Table S6**) since incomplete transcript coverage of some datasets resulted in the loss of some transcripts during merging (**Figure 1A**). The 174 transcripts are therefore present in each of the 20 compound treatments in the six datasets and cover all 103 time points in total. The 174-transcript rational selection database was used for building the predictive rational selection models (**Figure 1B**).

### Evaluating Different ML Algorithms on the Inclusive Database

**Figure 2:**
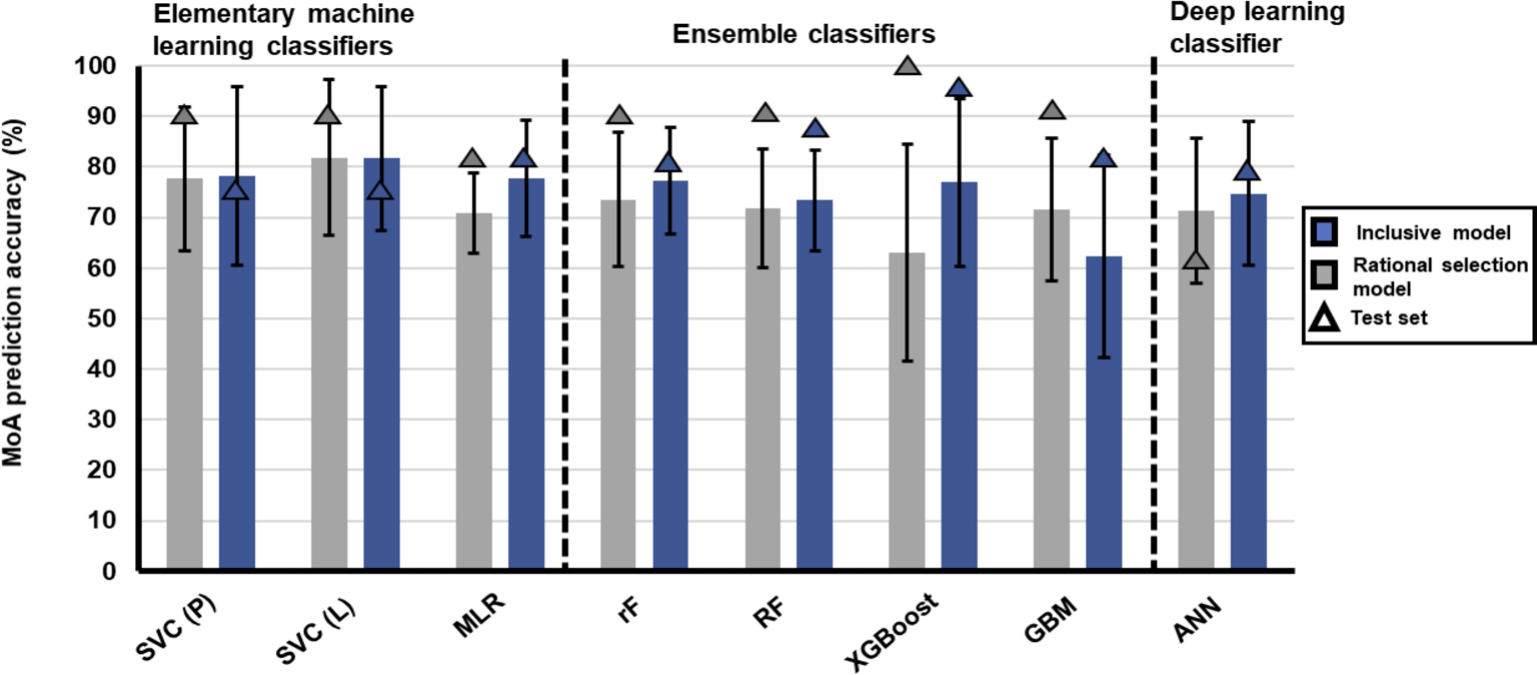
Robustness and accuracy of different ML algorithm’s ability to stratify treatments with similar MoA using either the 2463-transcript inclusive database or 174-transcript rational selection database. Different ML algorithms are grouped according to statistics-driven elementary ML, ensemble or deep learning classifiers. Algorithm classifiers were either trained on the 2463-transcript inclusive database (blue) or 174-transcript rational selected database (gray). Classifiers were hyperparameter tuned before undergoing 10-fold cross-validation. Bars indicate the average accuracy of the classifier obtained from 10-fold cross-validation on the training data and the error bars are the standard deviation of performance measures. Triangles indicate the accuracy of the classifier in stratifying the MoA of test data. Some ML algorithms had variations in R packages that could be used for model building, which were also interrogated. SVC, support vector classification with the1071 R package; P, polynomial; L, linear; MLR, multinomial logistic regression; rF, random forest with randomForest R package; RF, random forest with h2o R package; XGBoost, built with xgboost R package, GBM, gradient boosting machine; ANN, artificial neural network. MLR. RF GBM and ANN was built using h2o R package.

During the model building, inclusive ML models were trained using 80% of the randomly selected data points from the 2463-transcript inclusive database (defined as the training set) and hyperparameter tuning was applied to SVC, GBM, RF and ANN inclusive models (**Figure 1B** and **Supplementary Tables S4**, **S5**). Elementary ML models such as the polynomial and linear kernel SVCs displayed similar high model variability with accuracies of 78.2 ± 17.7% and 81.7 ± 14.2%, respectively, compared to MLR which had lower model variability with an accuracy of 77.8 ± 11.6% (**Figure 2**). The other SVC kernels trained on either the inclusive or rational database performed extremely poorly (**Supplementary Figure S3**) and were not further investigated.

The ensemble classifier set performed slightly worse with most of the models displaying accuracies between 73-77%, with GBM (h2o R package) the most inaccurate at 62%. The RF model built using the randomForest R package performed well at 77.3 ± 10.6% compared to MLR and the other RF model (h2o), which displayed less model variability (10%) but also lower accuracy (73%). Quite high model variability was observed (up to 20%) for the remaining ensemble classifiers. Within the deep learning classifier, the same large variability regarding accuracy was also observed for the h2o ANN algorithm (accuracy at 74.77 ± 14.17%).

During the evaluation of model performance on the test set, both the SVC algorithms performed poorer than their average accuracy achieved on the 10-fold cross validation, indicating that these models may have become overfitted to the training set and struggled in stratifying untrained data. However, all the other algorithms performed well with the test data. Both the MLR and RF (randomForest) algorithms displayed similar efficacy as evaluated by accuracy, model variability, and ability to perform on test data, therefore, these algorithms are useful to generate models on larger datasets that are informative to stratify compounds based on specific MoAs. Most of these algorithms generated models with high variability (>10%) in MoA stratification, possibly as a result of including transcripts that are ‘noisy’ and non-informative for MoA stratification as training features. Non-informative transcripts were subsequently excluded, and transcripts representative of a compound’s MoA were identified in the rational selection approach.

### Evaluating Different ML Algorithms on the Rational Selection Database

Rational selection models were generated using the same algorithms trained on 80% of the randomly selected data points from the 174-transcript rational selection database. Both polynomial and linear kernel for SVCs (**Figure 2**) showed high model variability and accuracies of 77 ± 14.3% and 81.8 ± 16.4%, respectively. By contrast, the other elementary ML model, MLR, although slightly less accurate at 70.9%, was very robust with very little model variability observed (± 8%) (**Figure 2**).

Within the ensemble classifier set, the majority of the models had fair accuracy ~73%, except for XGBoost at 63%. However, in all instances, high model variability was observed, ranging between 11-21%. The same large variability was observed for the deep learning classifier built using the h2o ANN algorithm (accuracy at 71.3 ± 14.3%). To evaluate the performance of the models on untrained data, the remaining 20% from the 174-transcript rational selection database used as a test set. From this, only the h2o ANN algorithm for deep learning performed poorly, indicating overfitting of the model to the training set due to the inability of the model to generalize and recognize patterns in untrained data (63%). Although ensemble classifiers did well in predicting the test set, these algorithms do not perform well during 10-fold cross validation as seen by the high model variability. Since this cross validation splits the training data to get a better estimate of the model’s accuracy, it revealed that these algorithms are not reliable when the number of samples are reduced.

Taken together, the MLR algorithm generated the most effective model for the 174-transcript rational selection database based on its’ accuracy, combined with low model variability and good performance on test data.

### Validation of Rational Gene Selection

**Figure 3:**
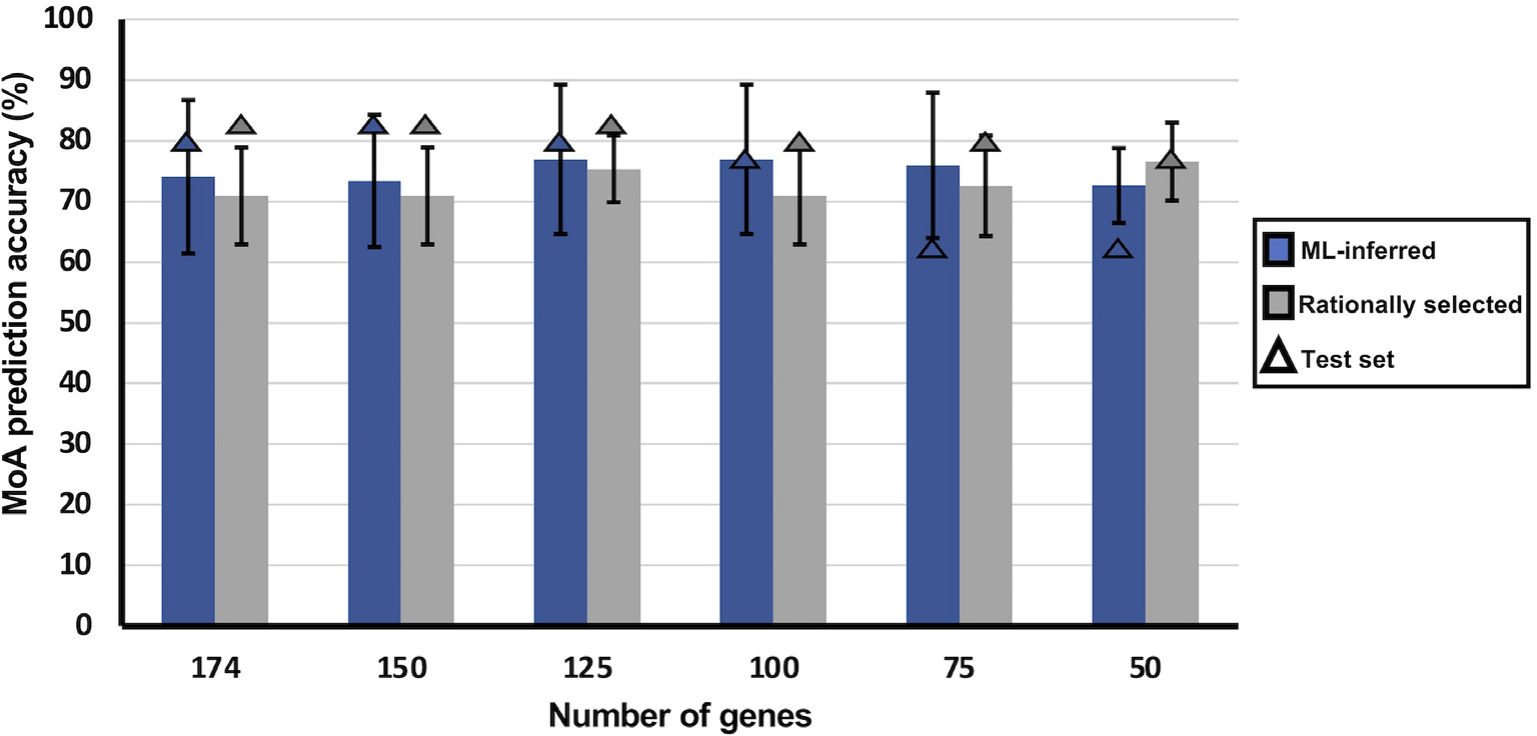
Influence of limiting the number of transcripts used as training features on MoA stratification of MLR models. MLR classifiers trained on either ML-inferred transcripts (blue) or on rationally selected transcripts (light gray). Using variable importance, transcripts were ranked according to their importance in making classification decisions for the MLR classifier. With the ranked transcripts a sliding gene-scale approach was applied where the top transcripts were used to make minimodels with each sequential model containing decreasing number of transcripts used to train the MLR classifier. Each minimodels underwent 10-fold cross-validation and was also assessed in the accuracy of MoA stratification on test data. Bars indicate average accuracy obtained from 10-fold cross-validation, and triangles indicate model accuracy on the untrained test set. Error bars indicates the standard deviation of the average accuracy.

To assess which gene selection approach was the best to identify transcripts representative of MoA, a sliding gene-scale method was applied, where smaller models (minimodels) are built with sequentially fewer transcripts as training features, to remove non-informative features and obtain the minimum transcript model (**Figure 3**). Transcripts used to generate these models were based on their ranked variable importance. The average MoA prediction accuracy of the rational selection minimodels was either maintained or increased >70%, even when lowering the number of training transcripts to only 50 (**Figure 3**). The model variability within the minimodels seems to decrease as the number of training transcripts are reduced, with the minimodel using the top 75 transcripts being an exception (76.0 ± 8.3%). Performance of these minimodels on the test set resulted in an accuracy of >75%.

The opposite is observed for the ML-inferred selection minimodels, where a general increasing trend in variability is observed in the minimodels with reduced transcripts as well as a decline in performance on test data, which indicates overfitting (**Figure 3**). The latter was confirmed by increased RMSE and log-loss values for the 50-transcript ML-inferred minimodel (**Supplementary Figure S5**), validated by a higher difference between R^2^ and Q^2^LOO (leave-one-out cross validation correlation coefficient) of the ML-inferred minimodels (**Supplementary Figure S5**). This again reaffirms that the transcripts from our rational selection approach are more suitable for MoA stratification than that of ML-inferred transcripts from the inclusive database. Although a gradual decline in performance on the test set is observed as the number of training transcripts is reduced for our rational selection minimodels (75%), it is not to the extent as that within the ML-inferred selection minimodels (63%).

Based on the test set performance, as well as accuracy, model variability and the least number of transcripts used for training, the rational selection 50-transcript minimodel (76.6 ± 6.4%) was identified as the optimal minimum number of transcripts for robust MoA stratification of compounds.

### 50 Biomarkers as Indicators of MoA

The 50-transcript minimodel from the rational selection MLR model was subsequently manually interrogated (**Table 1**). These top 50 transcripts are therefore defined as biomarkers that were identified from 14 of the 20 compounds, which account for 12 of the 15 MoAs of all the compounds in our database. Interestingly, some compounds contribute more to the overall biomarkers used in the MLR model than others. For example, from the artemisinin treatment only two biomarkers are utilized, whereas for the TSA treatment five biomarkers exist. In fact, the majority of the compounds that contribute to the overall biomarkers used by the model are inhibitors of proteins that serve important and global functions within a cell, such as kinases and deacetylases (**Table 1**). Of the top 50 biomarkers, 42% have putative protein products ascribed to them and 30% encode a novel protein with unknown function that may be involved in important cellular processes and make them useful for biomarkers in MoA stratification (**Table 1**). Of the top 50 biomarkers, 11 are annotated to be involved in ATP or DNA binding, and eight seem to be involved in translation and transcription processes within the parasite.

**Figure 4:**
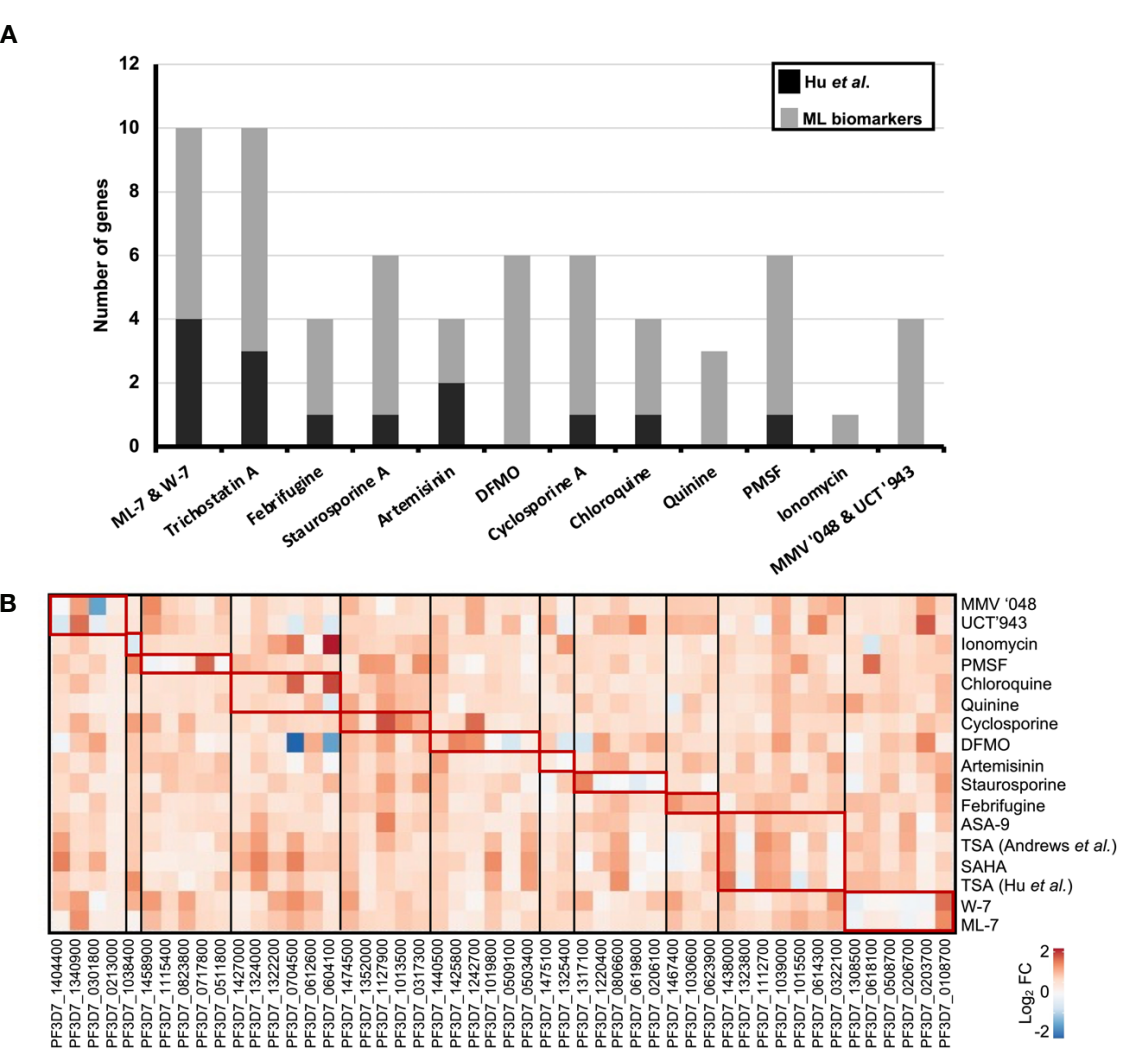
Novelty of 50 rationally selected biomarker transcripts expression patterns associated with similar MoA. **(A)** The top 50 biomarkers (gray) from the rational selection 50-transcript MLR minimodel were compared to DEGs associated to MoA as identified by (Hu et al., 2009) shown in black. Biomarker transcripts which were also identified for the same compound and MoA within these two studies are shown as stacked bars. **(B)** Correlation in gene expression of the 50 biomarkers between compounds within our database. Log_2_ fold change values for each transcript was extracted in all the compound treatments to plot the heatmap. Similar expression patterns (red blocks) are seen within compound treatments with similar MoA. The biomarkers are grouped according to the MoA (black blocks) they were identified from in **Table 1**.

To evaluate the novelty of the top 50 biomarkers associated with specific MoAs, we compared them to transcripts identified by Siwo et al. (2015) and Hu et al. (2009) as associated with specific MoAs (**Figure 4A**). No overlap was observed for the 50 biomarkers from our study compared to the Siwo dataset, possibly due to selection differences in DE cutoffs as the Siwo dataset defined DE as the top 100 up and down regulated genes for a treatment. Moreover, only 13% (14) of the biomarkers we identified associated with the same compound MoA in the Hu dataset. The Hu et al. dataset used a 3 fold-change cut-off to define DEGs, whereas we defined DEGs as those within the upper or lower 5^th^ percentile of gene expression, allowing us to identify more DEGs.

We next evaluated the expression profiles for each of the 50 biomarkers across all the individual compound treatments used within our original dataset (**Figure 4B**). A clear distinction was observed for each set of transcripts that describe the profile of a particular compound class, with little overlap between biomarkers for different compounds (**Table 1** and **Figure 4B**). Importantly, each of the biomarkers selected from the models are unique (**Table 1**). The unique profiles obtained is particularly distinct for compounds that are known to target single protein targets, where a specific fingerprint is obtained with a pronounced differential response (**Figure 4B**). This corresponds to these compounds affecting 10-20% of the genome as a result of inhibition of mechanisms that regulate *P. falciparum* asexual development (Hu et al., 2009). Not surprisingly, compounds such as chloroquine elicit a weaker overall DE response associated with their biomarkers, similar to previous reports for chloroquine and others that affect ~5% of the genome with low amplitude, albeit with reproducible specificity (Hu et al., 2009) (**Figure 4B**). The biomarkers are therefore unique to each compound treatment MoA and are able to distinguish compounds based on varied transcriptional responses.

### Biomarker Fingerprints Stratify Compounds Based on MoA

**Figure 5:**
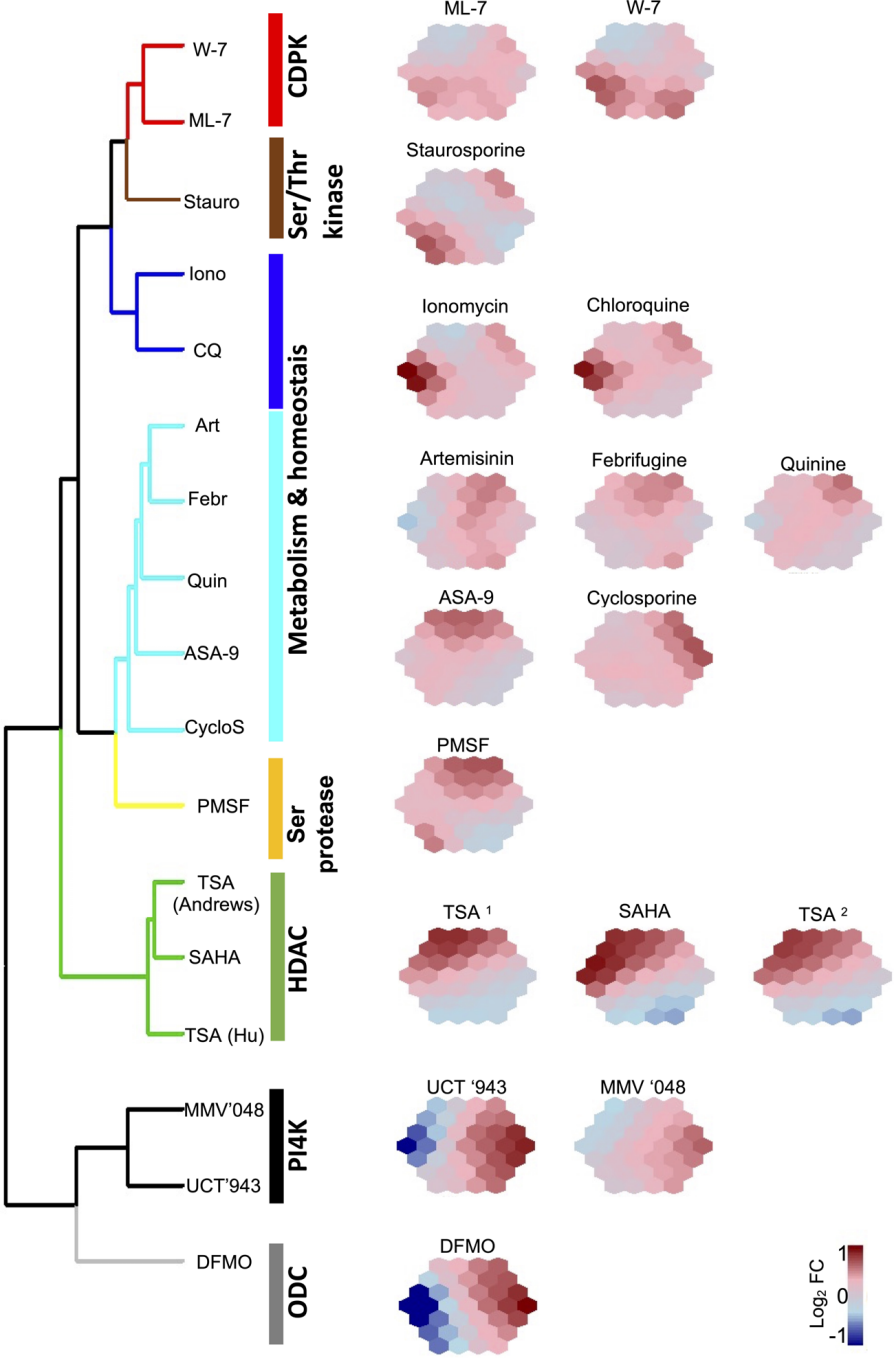
The ML defined biomarkers result in unique suprahexagonal chemo-transcriptomic fingerprints clustered per compound MoA. The dimensionality of expression profiles for the 50 biomarkers from various compound treatments were reduced using self-organizing maps (SOM) visualized as suprahexagonal chemo-transcriptomic fingerprints. Each hexagon within each suprahexagonal map defines a cluster of biomarkers colored according to log_2_ fold change (FC) expression values, and hierarchically clustered using Ward linkage on Euclidian distance of expression profiles. Known protein targets or biological processes affected by the compound treatments are indicated. CDPK, Ca^2+/^calmodulin-dependent protein kinase; Ser/Thr kinase, serine/threonine kinase; Ser protease, serine protease; HDAC, histone deacetylase; ODC, ornithine decarboxylase; Stauro, staurosporine; Iono, ionomycin; CQ, chloroquine; Art, artemisinin; Febr, febrifugine; Quin, quinine; ASA-9, 2-aminosuberic acid derivative; CylcoS, cyclosporine A; PMSF, phenylmethylsulfonyl fluoride; TSA, trichostatin A; SAHA; suberoylanilide hydroxamic acid; DFMO, difluoromethylornithine; TSA1, data from Hu et al. (2009); TSA2, data from Andrews et al. (2012).

We next interrogated the 50 biomarkers selected for their usefulness to stratify a compound’s MoA and generate a chemo-transcriptomic fingerprint associated with compound MoA classes. We generated self-organizing maps (SOMs) for each treatment (**Figure 5**), similar to those defined for metabolomic fingerprints (Allman et al., 2016). This revealed distinct fingerprints based on the expression profiles of the biomarkers for each of the treatments used in the ML model. Importantly, compounds with similar fingerprints group together, indicating similar MoA and supporting the specificity of the biomarkers identified (**Figure 5**). However, a number of compounds with less well-defined molecular targets, or where their MoA is ascribed to multiple biological processes being affected, cluster together (**Figure 5**, cluster ‘metabolism and homeostasis’). This includes chloroquine and artemisinin that generally affect hemoglobin catabolism or cause cellular damage due to radical formation, respectively (Hu et al., 2009; Klonis et al., 2013; Tilley et al., 2016; Xie et al., 2020). Additionally, the resolution of the chemo-genomic fingerprint was such that it could indicate the presence of subclasses: even though ionomycin (a Ca^2+-^binding ionophore) has a fingerprint clustered with that of chloroquine, it is still distinct (**Figure 5**).

The specificity of the chemo-genomic fingerprints is exemplified in instances where compounds with similar drug targets have the same biomarkers and as a result, similar chemo-transcriptomic fingerprints cluster together (**Figure 5**). The HDAC inhibitors TSA and suberoylanilide hydroxamic acid (SAHA, Vorinostat) have very similar fingerprints and cluster into the same group. These compounds are both hydroxymate-based inhibitors that target affect multiple stages of malaria parasites (Andrews et al., 2012; Coetzee et al., 2020) by inhibiting multiple HDACs, particularly HDAC1 (PF3D7_0925700) and HDA1 (PF3D7_147220) (Coetzee et al., 2020). The biomarker-based chemo-transcirptomic fingerprints are therefore able to classify these chemically-related compounds together based on similar drug targets. Moreover, derivatisation of the hydroxymate-based core to include 2-aminosuberic acid in compounds such as ASA-9 (Andrews et al., 2012) resulted in an individual fingerprint with similarity to the TSA and SAHA fingerprints, but that clusters with the less distinct group. It must be noted that the target of ASA-9 is not confirmed and the deviation therefore can indicate differentiation in MoA and possible off-target effects due the changes in the core structure of the comounds. This indicates that the biomarker-based chemo-transcriptomic fingerprints are sensitive enough to distinguish structural derivatisation from chemotype cores, whilst at the same time retain grouping within similar MoAs.

ML-7 and W-7 are both CDPK inhibitors (Hu et al., 2009) and these two compounds share biomarkers that result in similar fingerprints, distinct from others (**Figure 5**). Additionally, the lipid kinase (phosphatidylinositol 4-kinase, PI4K) inhibitors MMV’048 and UCT’943 are closely related 2-aminopyridine and 2-aminopyrazines, respectively (Paquet et al., 2017; Brunschwig et al., 2018; van der Watt et al., 2018). These two PI4K inhibitors also share biomarkers with the result that they have similar fingerprints. However, there is a distinct difference between the fingerprints for the CDPK and PI4K kinase inhibitors, implying that the ML model is able to distiguish different kinase inhibitors and is sensitive enough to stratify compounds even when they fall into a larger class of proposed drugs like kinase inhibitors. The model is therefore able to predict different outcomes on cellular signalling processes coordinated by different kinases.

Interestingly, 16 of the 50 biomarkers can also be associated with copy number variations (CNVs, nine biomarkers) or single nucleotide variations (SNVs, eight biomarkers) that typically results in resistance phenotypes of *P. falciparum* (Cowell et al., 2018). This information is used to predict possible drug targets or resistance mechanisms (Cowell et al., 2018). One of our biomarkers, *pf3D7_0108700* (encoding a putative secreted ookinete protein) produced CNVs for four different treatments (Cowell et al., 2018), hinting at a similar drug target or resistance mechanisms for these compounds. However, this gene is not part of the described resistome for the parasite (Cowell et al., 2018). Since none of the biomarker sets for a particular compound show major overlap in their entirety with the resistome, it is unclear whether those that are associated with CNVs or SNVs are predictive of resistance development or simply as a result of fitness costs due to other resistance-inducing mutations co-occurring.

### Validation of the Biomarker Selection

**Figure 6:**
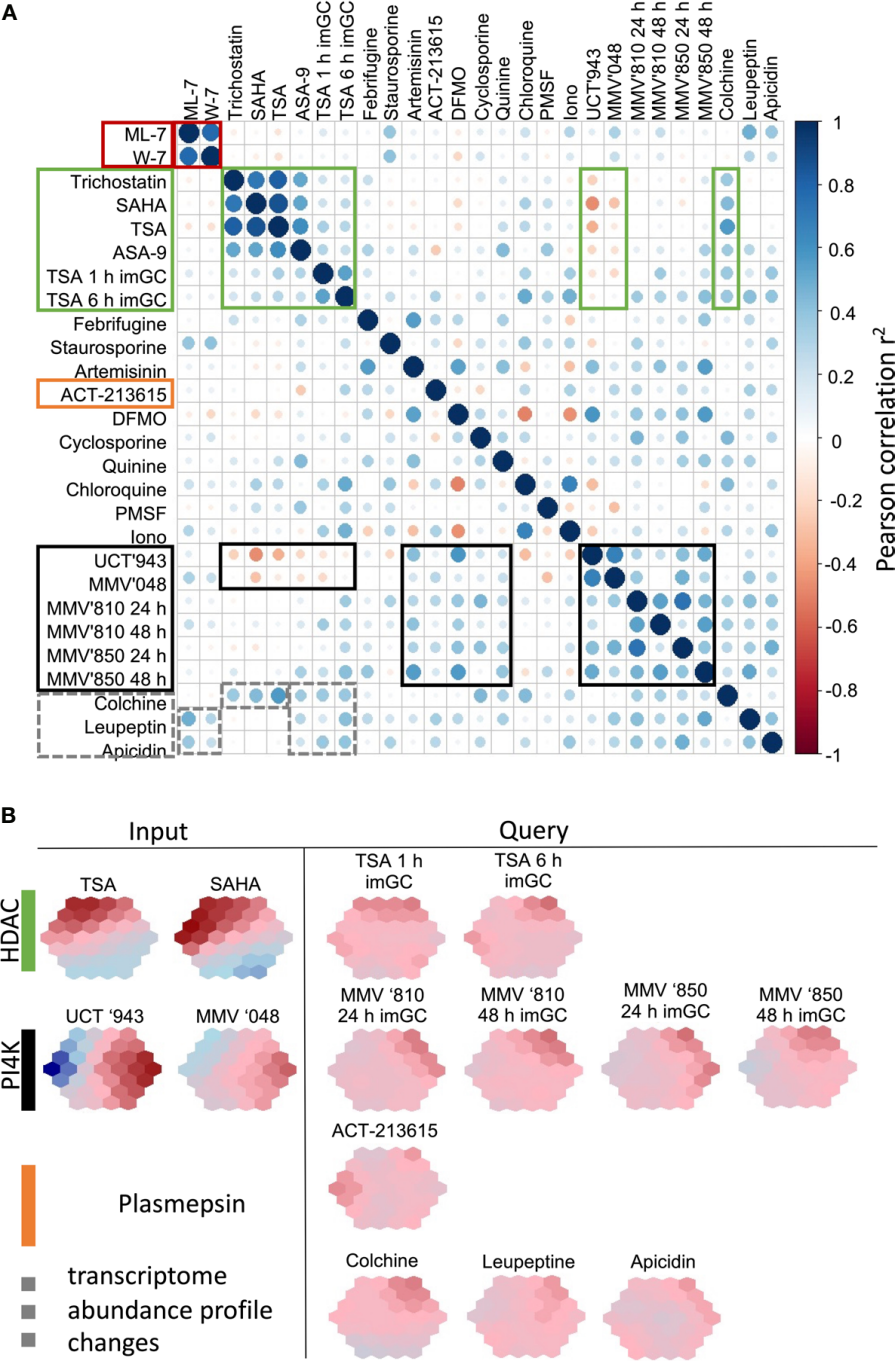
Performance of the biomarkers to stratify new compounds to MoA classes. **(A)** Correlation plots were generated from biomarker expression profiles for compounds within our database as well as new compounds (TSA, MMV666810 and MMV642850) treated on immature gametocytes (stage II/III) to assess the usefulness of the biomarkers on different life cycle stages of the parasite. Plots were visualized using corrplot based on Pearson correlations. Similar correlation patterns, shown in blocks, were observed for compound treatments with similar MoA. Areas of high correlation (positive or negative) are indicated in blocks for particular compound groups. **(B)** SOM suprahexagonal chemo-transcriptomic fingerprints for each of the new compounds included in the validation compared to example compounds within the same MoA class. TSA, Trichostatin A; MMV666810, MMV’810; MMV642850, MMV’850; im Gc, immature stage II/III gametocytes.

We subsequently set out to interrogate the performance of the biomarker fingerprints to be able to stratify new compounds into particular MoAs. To do this, we incorporated transcriptome data from compounds that were not included into the original ML model design. Several of the data used to interrogate the biomarkers showed clear correlation to compounds with defined MoA that was included to generate the model (**Figure 6**). However, compounds such as colchicine, leupeptin and apicidin have low DE amplitudes (Hu et al., 2009) and indeed does not show pronounced correlation to any other compounds, except for the expected overlap between the HDAC inhibitor apicidin and other HDAC inhibitors. Apicidin, together with the pan-protease inhibitor leupeptin also show correlation (r^2^<0.4) with the CDPK inhibitors and may be as a result of the pleotropic effects of these compounds that affect the asexual parasite’s transcriptional profile. Similarly, colchicine, as microtubule formation inhibitor, falls within this group and correlates also with the HDAC inhibitors (r^2 =^ 0.57, **Figure 6**).

ACT-213615 was identified as a food vacuole plasmepsin inhibitor (Brunner et al., 2012) and this compound’s fingerprint does not correspond to any of the classes of antimalarials incorporated in the model (**Figure 6**). With the poor correlations to the other datasets, it is therefore clear that this compound has a MoA dissimilar to any of the other control compounds. This indicates that the ML model and fingerprinting is able to identify compounds that do not overlap in target and MoA with other compounds and will be able to indicate new MoAs from this approach.

Lastly, we also asked if our biomarkers are robust enough to stratify compounds’ MoA when other life cycle stages are treated. We argued that at least some overlap should be visible within MoA classes particularly between the transcriptomes of asexual parasites and immature (stage II/III) gametocytes, since stage-specific transcriptome variation between these stages are not as pronounced as between asexual parasites and mature gametocytes (van Biljon et al., 2019). We therefore included data where immature gametocytes were treated with TSA (Ngwa et al., 2017), and a positive correlation (r^2^~0.3) was seen with the biomarker predictions from the asexual parasite data, resulting in a similar fingerprint for these compounds, although with a less pronounced amplitude in the immature gametocyte profiles (**Figure 6B**). All of these HDAC inhibitors, irrespective of life cycle stage, anticorrelated with the profiles of lipid kinase inhibitors, but did correlate with the colchicine profile, again confirming distinct changes in the transcriptome of *P. falciparum* that is conserved across asexual parasites and immature gametocytes.

This same grouping of PI4K inhibitors were seen when two other compounds, similar to MMV’048 and UCT’943, MMV666810 (MMV’810) and MMV642850 (MMV’850) was included. MMV’810 is a 2-aminopyridine derivative whereas MMV’850 falls within the imidazopyridazine class of kinase inhibitor scaffolds (van der Watt et al., 2018). Transcriptome profiles of immature gametocytes treated with MMV’810 and MMV’850 (van Biljon et al., submitted) again displays a distinct chemo-transcriptomic fingerprints, disconnected from that of HDAC inhibitors. The additional interrogation of the dynamics associated with the fingerprints for MMV’810 and ‘850 confirmed a rate of onset > 24 h, that is more pronounced for MMV’850 (**Figure 6B**).

The biomarkers are therefore accurate to classify compounds with unknown MoA into their own classes and robust enough to be used to evaluate compound fingerprints and MoA across multiple life cycle stages.

## Discussion

Although *P. falciparum* parasites stringently control their gene expression profiles during normal development and differentiation (Bozdech et al., 2003; van Biljon et al., 2019), several reports indicate that antimalarial compound treatment results in measurable changes in the transcriptome with an appreciable and individualized transcriptional signature (Hu et al., 2009; Siwo et al., 2015). Here, we report that such chemically-induced transcriptional responses can be used to create an informative ML model that is able to stratify antiplasmodial compounds to their respective MoA with robust accuracy. The ML model could be defined with a minimum set of biomarkers and these could ascribe a unique chemo-transcriptomic fingerprint for each compound. Moreover, compounds with similar fingerprints (and per implication MoAs) grouped together.

This is one of the first reports of the successful use of ML to stratify compounds based on their MoA using chemically-induced transcriptional profiles for *P. falciparum* parasites. Although GEPs provide a global overview of how the parasite is affected by a compound, they contain high dimensionality with irrelevant and/or redundant features that limit the efficiency and generalization of ML algorithms (Bolón-Canedo et al., 2015). We overcame this developmental challenge by applying a rational gene selection approach to reduce the dimensionality in the training features. This highlights the importance of training data resembling the data it will be tested on such that noisy input data is limited as this will influence the model’s prediction accuracy (Sanders and Saxe, 2017; Beam and Kohane, 2018; Pu et al., 2019). ML algorithms can additionally not necessarily account for biological significance and may assign significance to transcripts that are biologically irrelevant to the MoA and cell processes affected by the compound. Similar to studies in other systems (Zhao et al., 2010; Crawford and Greene, 2020), we see that prior biological information can and should therefore be used for feature selection to ensure the ML algorithms select features that are the best predictors relevant to the problem. Whilst the incorporation of networks or pathways describing relationships between genes are informative when using gene expression data, such information is often incomplete and lacking in the malaria field compared to other fields and does limit the approaches that can be taken. In line with strategies being applied in other disease areas like cancer (Zhao et al., 2010) integrating prior biological knowledge and insight about the disease indeed strenghtened the capabilities of ML algorithms use here to assign biologically relevant significance.

The limited features available in the transcriptome dataset for compound-treated *P. falciparum* parasites, resulted in MLR performing as the best ML algorithm to stratify the compounds’ MoA. This could be ascribed to MLR’s ability to use logistic regression and maximum likelihood rather than transforming data with ML algorithms like SVC and ANN. Interestingly, algorithms employing ensemble classifiers or deep learning showed high variability within them compared to MLR despite such techniques typically displaying good performance for multiclassification problems utilizing gene expression in other cancer studies (Khan et al., 2001; Kim et al., 2019). Techniques such as ANN and ensemble classifiers are more adapted to handle ‘big data’ such as CMap, which contains about 1.5 M expression profiles of over 5000 compounds treated on different cancer cell types (Sirci et al., 2016).

The resultant MLR model could successfully identify a limited set of 50 biomarkers that are informative to stratify the different compounds’ transcriptional response from each other. Although 50 biomarkers for MoA stratification may be considered low, it is similar to the 70 transcripts that were identified as informative biomarkers for MoA classification from the *Mycobacterium tuberculosis* transcriptome (Murima et al., 2013). The selected biomarkers are all unique and continually DE throughout a compound’s treatment. This confirms that the parasite’s chemically-induced transcriptome response is individualized, robust and directed, a characteristic not limited to this protist, but also evidently clear for *M. tuberculosis* (Murima et al., 2013), with deregulation of the transcriptome evident over and above the general, typically tight regulation of transcriptomes (Bozdech et al., 2003; van Biljon et al., 2019). The two genomes of these organisms have a similar size, and the biomarker pools therefore present only ~1% of their transcriptomes, attesting to the power of ML to identify biomarkers for the most informative, condensed subset of the transcriptome.

ML modelling and biomarker selection successfully described chemo-transcriptomic fingerprints for each compound investigated, and classified compounds together based on similar MoA. Importantly, the clustering was correlated directly with the specific molecular process or protein targeted and was not seemingly influenced by differences in chemotype, unless a derivative of the chemotype resulted in proposed off-target effects e.g., ASA-9 not clustering with other HDAC inhibitors. Moreover, compounds with clear molecular targets e.g., PI4K inhibitors, have more pronounced and specific fingerprints, in contrast to metabolic profiling where pronounced fingerprints were only obtained for compounds affecting broader biological systems (Allman et al., 2016). This reflects increased sensitivity and specificity of chemo-transcriptomic fingerprint profiling and indicate that the latter may be a useful tool to completement metabolic profiling in cases where undefined metabolic fingerprints are observed. The ability of chemo-transcriptomic fingerprinting to also distinguish different kinase inhibitors like PI4K and CDPK inhibitors from each other, attests to its sensitivity to allow distinction between compounds that target different signaling pathways in the parasite, although both ultimately leads to transcriptional deregulation.

The biomarkers displayed robustness regarding differences due to life cycle stage evaluated and was still able to classify compounds into the same classes even though a weaker overall response was observed in immature gametocytes compared to asexual parasites. Since the majority of biomarkers selected in the ML models have undescribed functions, it is intriguing that these are then still informative to chemo-transcriptomic fingerprinting and implicates their involvement in shared biological processes or responses across asexual parasites and immature gametocytes. As more data is generated for additional life cycle stages like gametocytes, the ML model can be further optimized and expanded to handle stage-specific variation in the *Plasmodium* transcriptome.

The ML model and biomarkers are immediately useful in e.g. a focused-array format to allow the rapid evaluation of new antimalarial compounds (and their derivatives) within a H2L or LO program. This should provide information on new compounds’ MoA that can either overlap with existing compounds/drugs or to be unique. Additionally, any deviation from this due to structural changes of the compounds could be rapidly detected. The benefit of ML models lie in the fact that they are dynamic and can be updated as new data is generated and incorporated. In each such instance, the models become more powerful in their predictive and classifying nature, improving on issues such as class-bias. The model could therefore be extended in the next phase by including data on pre-clinical and clinical candidates within the MMV pipeline, to allow comparison with the metabolomic fingerprints for these frontrunners (Allman et al., 2016).

In summary, our study has identified unique 50 biomarkers capable of stratifying antimalarial compounds based on their MoA. This was achieved using a MLR model that is stable, specific and accurate. With this, we show that chemo-transcriptomic fingerprints exist for individual compounds and can classify compounds with similar MoA together. This provides a new tool in the toolkit to describe antimalarial drug MoA to accelerate the drug discovery process by rapidly providing data to guide H2L and LO strategies.

## Data Availability Statement

The datasets used in this study can be found at Gene Expression Omnibus using accession numbers provided in the **Supplementary Material**. All scripts and data are available at https://github.com/Ash-bot/MoA-Stratification-model-scripts-and-data.

## Author Contributions

AvH performed the research with RvW. LMB conceptualized the study and wrote the paper with AvH. All authors contributed to the paper and approved the submitted version of the paper.

## Funding

This work was supported by the South African Department of Science and Innovation and National Research Foundation South African Research Chairs Initiative Grant (L-MB UID: 84627).

## Conflict of Interest

The authors declare that the research was conducted in the absence of any commercial or financial relationships that could be construed as a potential conflict of interest.

## Abbreviations

ANN, artificial neural network; CDPK, Ca^2+^/calmodulin-dependent protein kinase; CMap, Connectivity Map; CNV, copy number variants; DE, differentially expressed; DEG, differentially expressed gene; GBM, gradient boosting machine; GEP, gene expression profile; GEO, gene expression omnibus; HDAC, histone deacetylase; H2L, hit-to-lead; LO, lead optimization; LOOCV, leave-one-out cross validation; ML, machine learning; MLR, multinomial logistic regression; MMV, medicines for malaria venture; MoA, mode of action; PI4K , phosphatidylinositol 4-kinase; RF, random forest; RMSE, root mean squared error; SOM, self-organizing maps; SNV, single nucleotide variants; SVC, support vector classification; TSA, trichostatin A; Q^2^LOO, leave-one-out cross validation correlation coefficient.

## References

[B1] Abraham M. Gagaring K. Marino M. L. Vanaerschot M. Plouffe D. Calla J.. (2020). Probing the Open Global Health Chemical Diversity Library for Multistage-Active Starting Points for Next-Generation Antimalarials. ACS Infect. Dis. 6 (4), 613–628. 10.1021/acsinfecdis.9b00482 32078764 PMC7155171

[B2] Allman E. L. Painter H. J. Samra J. Carrasquilla M. Llinás M. (2016). Metabolomic Profiling of the Malaria Box Reveals Antimalarial Target Pathways. Antimicrob. Agents Chemother. 60, 6635–6649. 10.1128/AAC.01224-16 27572391 PMC5075069

[B3] Andrews K. T. Gupta A. P. Tran T. N. Fairlie D. P. Gobert G. N. Bozdech Z. (2012). Comparative Gene Expression Profiling of P. Falciparum Malaria Parasites Exposed to Three Different Histone Deacetylase Inhibitors. PloS One 7, e31847. 10.1371/journal.pone.0031847 22384084 PMC3288058

[B4] Beam A. L. Kohane I. S. (2018). Big Data and Machine Learning in Health Care. Jama 319, 1317–1318. 10.1001/jama.2017.18391 29532063

[B5] Birkholtz L.-M. Coetzer T. L. Mancama D. Leroy D. Alano P. (2016). Discovering New Transmission-Blocking Antimalarial Compounds: Challenges and Opportunities. Trends Parasitol. 32, 669–681. 10.1016/j.pt.2016.04.017 27209388

[B6] Bolón-Canedo V. Sánchez-Maroño N. Alonso-Betanzos A. (2015). Feature Selection for High-Dimensional Data (Switzerland: Springer International Publishing).

[B7] Bozdech Z. Llinás M. Pulliam B. L. Wong E. D. Zhu J. Derisi J. L. (2003). The Transcriptome of the Intraerythrocytic Developmental Cycle of Plasmodium Falciparum. PloS Biol. 1, E5. 10.1371/journal.pbio.0000005 12929205 PMC176545

[B8] Brownlee J. (2016). Machine Learning Mastery With R: Get Started, Build Accurate Models and Work Through Projects Step-by-Step (Melbourne, Australia: Machine Learning Mastery).

[B9] Brunner R. Aissaoui H. Boss C. Bozdech Z. Brun R. Corminboeuf O.. (2012). Identification of a New Chemical Class of Antimalarials. J. Infect. Dis. 206, 735–743. 10.1093/infdis/jis418 22732921

[B10] Brunschwig C. Lawrence N. Taylor D. Abay E. Njoroge M. Basarab G. S.. (2018). UCT943, a Next-Generation Plasmodium Falciparum Pi4k Inhibitor Preclinical Candidate for the Treatment of Malaria. Antimicrob. Agents Chemothe. 62 (9), e00012–18. 10.1128/AAC.00012-18 PMC6125526 29941635

[B11] Burrows J. N. Duparc S. Gutteridge W. E. Van Huijsduijnen R. H. Kaszubska W. Macintyre F.. (2017). New Developments in Anti-Malarial Target Candidate and Product Profiles. Malaria J. 16, 26. 10.1186/s12936-016-1675-x PMC5237200 28086874

[B12] Cawley G. C. Talbot N. L. Girolami M. (2007). “Sparse Multinomial Logistic Regression Via Bayesian L1 Regularisation”, in Advances in Neural Information Processing Systems (MIT Press), 209–216.

[B13] Cheemadan S. Ramadoss R. Bozdech Z. (2014). Role of Calcium Signaling in the Transcriptional Regulation of the Apicoplast Genome of Plasmodium Falciparum. BioMed. Res. Int. 2014, 869401. 10.1155/2014/869401 PMC4022301 24877144

[B14] Chen T. He T. Benesty M. Khotilovich V. Tang Y. Cho H.. (2019). Xgboost: Extreme Gradient Boosting.

[B15] Coetzee N. Von Grüning H. Opperman D. van der Watt M. Reader J. Birkholtz L.-M. (2020). Epigenetic Inhibitors Target Multiple Stages of Plasmodium Falciparum Parasites. Sci. Rep. 10, 1–11. 10.1038/s41598-020-59298-4 32047203 PMC7012883

[B16] Cowell A. N. Istvan E. S. Lukens A. K. Gomez-Lorenzo M. G. Vanaerschot M. Sakata-Kato T.. (2018). Mapping the Malaria Parasite Druggable Genome by Using In Vitro Evolution and Chemogenomics. Science 359, 191–199. 10.1126/science.aan4472 29326268 PMC5925756

[B17] Crawford J. Greene C. S. (2020). Incorporating Biological Structure Into Machine Learning Models in Biomedicine. Curr. Opin. Biotechnol. 63, 126–134. 10.1016/j.copbio.2019.12.021 31962244 PMC7308204

[B18] Creek D. J. Chua H. H. Cobbold S. A. Nijagal B. Macrae J. I. Dickerman B. K.. (2016). Metabolomics-Based Screening of the Malaria Box Reveals Both Novel and Established Mechanisms of Action. Antimicrob. Agents Chemother. 60, 6650–6663. 10.1128/AAC.01226-16 27572396 PMC5075070

[B19] Delves M. J. (2012). *Plasmodium* Cell Biology Should Inform Strategies Used in the Development of Antimalarial Transmission-Blocking Drugs. Future Med. Chem. 4, 2251–2263. 10.4155/fmc.12.182 23234549

[B20] Delves M. Lafuente-Monasterio M. J. Upton L. Ruecker A. Leroy D. Gamo F. J.. (2019). Fueling Open Innovation for Malaria Transmission-Blocking Drugs: Hundreds of Molecules Targeting Early Parasite Mosquito Stages. Front. Microbiol. 10, 2134. 10.3389/fmicb.2019.02134 31572339 PMC6753678

[B21] Delves M. J. Miguel-Blanco C. Matthews H. Molina I. Ruecker A. Yahiya S.. (2018). A High Throughput Screen for Next-Generation Leads Targeting Malaria Parasite Transmission. Nat. Commun. 9, 3805. 10.1038/s41467-018-05777-2 30228275 PMC6143625

[B22] Fang H. Gough J. (2014). Suprahex: An R/Bioconductor Package for Tabular Omics Data Analysis Using a Supra-Hexagonal Map. Biochem. Biophys. Res. Commun. 443, 285–289. 10.1016/j.bbrc.2013.11.103 24309102 PMC3905187

[B23] Gamo F.-J. Sanz L. M. Vidal J. De Cozar C. Alvarez E. Lavandera J.-L.. (2010). Thousands of Chemical Starting Points for Antimalarial Lead Identification. Nature 465, 305. 10.1038/nature09107 20485427

[B24] Gulati S. Ekland E.h. Ruggles K.v. Chan R.b. Jayabalasingham B. Zhou B.. (2015). Profiling the Essential Nature of Lipid Metabolism in Asexual Blood and Gametocyte Stages of Plasmodium Falciparum. Cell Host Microbe 18, 371–381. 10.1016/j.chom.2015.08.003 26355219 PMC4567697

[B25] Hizukuri Y. Sawada R. Yamanishi Y. (2015). Predicting Target Proteins for Drug Candidate Compounds Based on Drug-Induced Gene Expression Data in a Chemical Structure-Independent Manner. BMC Med. Genomics 8, 82. 10.1186/s12920-015-0158-1 26684652 PMC4683716

[B26] Hu G. Cabrera A. Kono M. Mok S. Chaal B. K. Haase S.. (2009). Transcriptional Profiling of Growth Perturbations of the Human Malaria Parasite Plasmodium Falciparum. Nat. Biotechnol. 28, 91. 10.1038/nbt.1597 20037583

[B27] Isik Z. Baldow C. Cannistraci C. V. Schroeder M. (2015). Drug Target Prioritization by Perturbed Gene Expression and Network Information. Sci. Rep. 5, 17417. 10.1038/srep17417 26615774 PMC4663505

[B28] Iwata M. Sawada R. Iwata H. Kotera M. Yamanishi Y. (2017). Elucidating the Modes of Action for Bioactive Compounds in a Cell-Specific Manner by Large-Scale Chemically-Induced Transcriptomics. Sci. Rep. 7, 40164. 10.1038/srep40164 28071740 PMC5223214

[B29] Josling G. A. Williamson K. C. Llinás M. (2018). Regulation of Sexual Commitment and Gametocytogenesis in Malaria Parasites. Annu. Rev. Microbiol. 72, 501–519. 10.1146/annurev-micro-090817-062712 29975590 PMC7164540

[B30] Keshavarzi Arshadi A. Salem M. Collins J. Yuan J. S. Chakrabarti D. (2019). Deepmalaria: Artificial Intelligence Driven Discovery of Potent Antiplasmodials. Front. Pharmacol. 10, 1526. 10.3389/fphar.2019.01526 32009951 PMC6974622

[B31] Khan J. Wei J. S. Ringnér M. Saal L. H. Ladanyi M. Westermann F.. (2001). Classification and Diagnostic Prediction of Cancers Using Gene Expression Profiling and Artificial Neural Networks. Nat. Med. 7, 673–679. 10.1038/89044 11385503 PMC1282521

[1366] Kim B.-H. Yu K. Lee P. C. W. (2019). Cancer Classification of Single-Cell Gene Expression Data by Neural Network. Bioinformatics 36 (5), 1360–. 10.1093/bioinformatics/btz772 31603465

[B33] Klonis N. Creek D. J. Tilley L. (2013). Iron and Heme Metabolism in Plasmodium Falciparum and the Mechanism of Action of Artemisinins. Curr. Opin. Microbiol. 16, 722–727. 10.1016/j.mib.2013.07.005 23932203

[B34] Kuhn M. Wing J. Weston S. Williams A. Keefer C. Engelhardt A.. (2019). Caret: Classification and Regression Training.

[B35] LeDell E. Gill N. Aiello S. Fu A. Candel A. Click C.. (2019). H2o: R Interface for ‘H2o’.

[B36] Liaw A. Wiener M. (2002). Classification and Regression by Randomforest. R News 2, 18–22.

[B37] Melas I. N. Kretsos K. Alexopoulos L. G. (2013). Leveraging Systems Biology Approaches in Clinical Pharmacology. Biopharm. Drug Dispos. 34, 477–488. 10.1002/bdd.1859 23983165 PMC4034589

[B38] Meyer D. Dimitriadou E. Hornik K. Weingessel A. Leisch F. (2019). E1071: Misc Functions of the Department of Statistics, Probability Theory Group (Formerly: E1071), Tu Wien.

[B39] Miguel-Blanco C. Molina I. Bardera A. I. Díaz B. De Las Heras L. Lozano S.. (2017). Hundreds of Dual-Stage Antimalarial Molecules Discovered by a Functional Gametocyte Screen. Nat. Commun. 8, 1–10. 10.1038/ncomms15160 28513586 PMC5442318

[B40] Moreira J. Carvalho A. Horvath T. (2018). A General Introduction to Data Analytics (United States of America: John Wiley & Sons).

[B41] Motoda H. Liu H. (2002). Feature Selection, Extraction and Construction. Vol. 5 (Taiwan: Communication of IICM (Institute of Information and Computing Machinery), 67–72.

[B42] Murima P. De Sessions P. F. Lim V. Naim A. N. M. Bifani P. Boshoff H. I. M.. (2013). Exploring the Mode of Action of Bioactive Compounds by Microfluidic Transcriptional Profiling in Mycobacteria. PloS One 8, e69191. 10.1371/journal.pone.0069191 23935951 PMC3729944

[B43] Murtagh F. Legendre P. (2014). Ward’s Hierarchical Agglomerative Clustering Method: Which Algorithms Implement Ward’s Criterion? J. Classifi. 31, 274–295. 10.1007/s00357-014-9161-z

[B44] Ngwa C. J. Kiesow M. J. Orchard L. M. Farrukh A. Llinás M. Pradel G. (2019). The G9a Histone Methyltransferase Inhibitor BIX-01294 Modulates Gene Expression During Plasmodium Falciparum Gametocyte Development and Transmission. Int. J. Mol. Sci. 20, 5087. 10.3390/ijms20205087 PMC6829282 31615031

[B45] Ngwa C. J. Kiesow M. J. Papst O. Orchard L. M. Filarsky M. Rosinski A. N.. (2017). Transcriptional Profiling Defines Histone Acetylation as a Regulator of Gene Expression During Human-to-Mosquito Transmission of the Malaria Parasite Plasmodium Falciparum. Front. Cell. Infect. Microbiol. 7, 320. 10.3389/fcimb.2017.00320 28791254 PMC5522858

[B46] Pabon N. A. Xia Y. Estabrooks S. K. Ye Z. Herbrand A. K. Süß E.. (2018). Predicting Protein Targets for Drug-Like Compounds Using Transcriptomics. PloS Comput. Biol. 14, e1006651. 10.1371/journal.pcbi.1006651 30532261 PMC6300300

[B47] Paquet T. Le Manach C. Cabrera D. G. Younis Y. Henrich P. P. Abraham T. S.. (2017). Antimalarial Efficacy of MMV390048, an Inhibitor of Plasmodium Phosphatidylinositol 4-Kinase. Sci. Trans. Med. 9 (387), eaad9735. 10.1126/scitranslmed.aad9735 PMC5731459 28446690

[B48] Plouffe D. Brinker A. Mcnamara C. Henson K. Kato N. Kuhen K.. (2008). In Silico Activity Profiling Reveals the Mechanism of Action of Antimalarials Discovered in a High-Throughput Screen. Proc. Natl. Acad. Sci. U. S. A. 105, 9059–9064. 10.1073/pnas.0802982105 18579783 PMC2440361

[B49] Pu Y. Apel D. B. Liu V. Mitri H. (2019). Machine Learning Methods for Rockburst Prediction-State-of-the-Art Review. Int. J. Min. Sci. Technol. 29, 565–570. 10.1016/j.ijmst.2019.06.009

[B50] Quancard J. Bach A. Cox B. Craft R. Finsinger D. Gueret S. M.. (2021). The European Federation for Medicinal Chemistry and Chemical Biology (Efmc) Best Practice Initiative: Phenotypic Drug Discovery. ChemMedChem 16 (11), 1737–1740. 10.1002/cmdc.202100041 33825353

[B51] Reader J. van der Watt M. E. Taylor D. Le Manach C. Mittal N. Ottilie S.. (2021). Multistage and Transmission-Blocking Targeted Antimalarials Discovered From the Open-Source Mmv Pandemic Response Box. Nat. Commun. 12, 269. 10.1038/s41467-020-20629-8 33431834 PMC7801607

[B52] Ritchie M. E. Phipson B. Wu D. Hu Y. Law C. W. Shi W.. (2015). Limma Powers Differential Expression Analyses for RNA-Sequencing and Microarray Studies. Nucleic Acids Res. 43, e47. 10.1093/nar/gkv007 25605792 PMC4402510

[B53] Sanders H. Saxe J. (2017). Garbage in, Garbage Out: How Purportedly Great ML Models Can Be Screwed Up by Bad Data. Proc. Blackhat 2017.

[B54] Sawada R. Iwata M. Tabei Y. Yamato H. Yamanishi Y. (2018). Predicting Inhibitory and Activatory Drug Targets by Chemically and Genetically Perturbed Transcriptome Signatures. Sci. Rep. 8, 156. 10.1038/s41598-017-18315-9 29317676 PMC5760621

[B55] Schratz P. Muenchow J. Iturritxa E. Richter J. Brenning A. (2019). Hyperparameter Tuning and Performance Assessment of Statistical and Machine-Learning Algorithms Using Spatial Data. Ecol. Modell. 406, 109–120. 10.1016/j.ecolmodel.2019.06.002

[B56] Simko T. (2017). R Package “Corrplot”: Visualization of a Correlation Matrix.

[B57] Sirci F. Napolitano F. Di Bernardo D. (2016). Computational Drug Networks: A Computational Approach to Elucidate Drug Mode of Action and to Facilitate Drug Repositioning for Neurodegenerative Diseases. Drug Discovery Today: Dis. Models 19, 11–17. 10.1016/j.ddmod.2017.04.004

[B58] Siwo G. H. Smith R. S. Tan A. Button-Simons K. A. Checkley L. A. Ferdig M. T. (2015). An Integrative Analysis of Small Molecule Transcriptional Responses in the Human Malaria Parasite Plasmodium Falciparum. BMC Genomics 16, 1030. 10.1186/s12864-015-2165-1 26637195 PMC4670519

[B59] Tan M. Özgül O. F. Bardak B. Ekşioğlu I. Sabuncuoğlu S. (2018). Drug Response Prediction by Ensemble Learning and Drug-Induced Gene Expression Signatures. Genomics 111 (5), 1078–1088. 10.1016/j.ygeno.2018.07.002 31533900

[B60] Tilley L. Straimer J. Gnädig N. F. Ralph S. A. Fidock D. A. (2016). Artemisinin Action and Resistance in Plasmodium Falciparum. Trends Parasitol. 32, 682–696. 10.1016/j.pt.2016.05.010 27289273 PMC5007624

[B61] Tulloch L. B. Menzies S. K. Coron R. P. Roberts M. D. Florence G. J. Smith T. K. (2018). Direct and Indirect Approaches to Identify Drug Modes of Action. IUBMB Life 70, 9–22. 10.1002/iub.1697 29210173

[B62] van Biljon R. Van Wyk R. Painter H. J. Orchard L. Reader J. Niemand J.. (2019). Hierarchical Transcriptional Control Regulates Plasmodium Falciparum Sexual Differentiation. BMC Genomics 20, 920. 10.1186/s12864-019-6322-9 31795940 PMC6889441

[B63] van Brummelen A. C. Olszewski K. L. Wilinski D. Llinas M. Louw A. I. Birkholtz L. M. (2008). Co-Inhibition of Plasmodium Falciparum S-adenosylmethionine Decarboxylase/Ornithine Decarboxylase Reveals Perturbation-Specific Compensatory Mechanisms by Transcriptome, Proteome, and Metabolome Analyses. J. Biol. Chem. 284, 4635–4646. 10.1074/jbc.M807085200 19073607 PMC3783055

[B64] van der Watt M. E. Reader J. Churchyard A. Nondaba S. H. Lauterbach S. B. Niemand J.. (2018). Potent Plasmodium Falciparum Gametocytocidal Compounds Identified by Exploring the Kinase Inhibitor Chemical Space for Dual Active Antimalarials. J. Antimicrob. Chemother. 73, 1279–1290. 10.1093/jac/dky008 29420756

[B65] Verlinden B. K. Louw A. I. Birkholtz L.-M. (2016). Resisting Resistance: Is There a Solution for Malaria? Expert Opin. Drug Discov. 11 (4), 395–406. 10.1517/17460441.2016.1154037 26926843

[B66] Wickham H. (2016). Ggplot2: Elegant Graphics for Data Analysis. (Springer-Verlag New York).

[B67] Woo J. H. Shimoni Y. Yang W. S. Subramaniam P. Iyer A. Nicoletti P.. (2015). Elucidating Compound Mechanism of Action by Network Perturbation Analysis. Cell 162, 441–451. 10.1016/j.cell.2015.05.056 26186195 PMC4506491

[B68] World Health Organization (2019). The “World Malaria Report 2019” at a Glance. (Geneva, Switzerland: World Health Organization).

[B69] Xie L. He S. Song X. Bo X. Zhang Z. (2018). Deep Learning-Based Transcriptome Data Classification for Drug-Target Interaction Prediction. BMC Genomics 19, 667–667. 10.1186/s12864-018-5031-0 30255785 PMC6156897

[B70] Xie S. C. Ralph S. A. Tilley L. (2020). K13, the Cytostome, and Artemisinin Resistance. Trends Parasitol. 36 (6), 533–544. 10.1016/j.pt.2020.03.006 32359872

[B71] Yang T. Ottilie S. Istvan E. S. Godinez-Macias K. P. Lukens A. K. Baragaña B. (2021). Malda, Accelerating Malaria Drug Discovery. Trends Parasitol. 37 (6), 493–507. 10.1016/j.pt.2021.01.009 33648890 PMC8261838

[B72] Zhang Y. Wong Y. S. Deng J. Anton C. Gabos S. Zhang W.. (2016). Machine Learning Algorithms for Mode-of-Action Classification in Toxicity Assessment. BioData Min. 9, 19. 10.1186/s13040-016-0098-0 27182283 PMC4866020

[B73] Zhao C. Bittner M. L. Chapkin R. S. Dougherty E. R. (2010). Characterization of the Effectiveness of Reporting Lists of Small Feature Sets Relative to the Accuracy of the Prior Biological Knowledge. Cancer Inf. 9, CIN.S4020. 10.4137/CIN.S4020 PMC2865771 20458361

