# Supplementary material for: Machine Learning Uses Chemo-Transcriptomic Profiles to Stratify Antimalarial Compounds With Similar Mode of Action
Source: Front Cell Infect Microbiol. 2021 Jun 29;11:688256. doi: 10.3389/fcimb.2021.688256 (PMC8277430; doi:10.3389/fcimb.2021.688256)
Supplement: Supplementary file 1 [file DataSheet_1.pdf]

Supplementary Material

Section 1: Supplementary Figures and Tables

1 Supplementary Figures and Tables

Multiple drug treated *P. falciparum* GEPs were downloaded (Supplementary Table S1) and assessed for their quality and whether the datasets fulfilled the filtering criteria (Supplementary Table S2) we developed. The accepted datasets (Supplementary Table S3) that fulfilled these criteria formed our database for model building. Before model training, the database needed to be pre-processed and normalized, hence cyclic loess normalization (Supplementary Figure S1) was implemented as it allowed for proper comparison between the accepted datasets. Two databases was generated in parallel, one containing all 2463-genes named the inclusive database, while the other database, named the rational selection database consisted of the 174-genes identified (Supplementary Table S6) through our rational gene selection (Supplementary Figure S2). MLR, SVC, RF, GBM and ANN models were built from each respective database. During model training the hyperparameter ranges (Supplementary Table S4) were assessed to determine the optimal hyperparameters that resulted in a model architecture which had higher accuracy in predicting antiplasmodial MoA from transcriptomic data. The optimal hyperparameters for each multiclassification algorithm was determined (Supplementary Table S5) and used to create MoA prediction models. These resultant models were assessed through cross-validation and accuracy in predicting the MoA of the test set (Supplementary Figure S3). SVC models using sigmoid and radial kernels performed extremely poorly with regards to accuracy and were excluded from further analysis. A sliding gene-scale approach was conducted using the two best performing algorithms, MLR (built using h2o R package) and RF (built using randomForest R package). To assess which gene selection approach identified the best predictive genes for MoA stratification, two models were generated for each algorithm in the sliding gene-scale approach, one trained on ML-inferred genes and another on the rationally selected genes (Supplementary Figure S4). Of the two algorithms, MLR models had considerably more accurate and stable models less prone to overfitting compared to respective RF models. To further validate overfitting within the ML-inferred minimodels compared to rationally selected minimodels, leave-one-out cross validation (LOOCV) was also conducted and the root mean squared error (RMSE), average log-loss and LOOCV correlation coefficient (Q^2LOO) was calculated for each minimodel (Supplementary Figure S5). Of the top 50 biomarker genes identified from the most robust MLR minimodel, 16 were also present as genes previously associated with copy number variations (CNVs) or single nucleotide variations (SNVs) resulting in resistance phenotypes after such mutant generation of *P. falciparum* due to drug pressure (Supplementary Table S7) (Cowell *et al*., 2018).

1.1 Supplementary Tables

Table S1: *P. falciparum* compound treated GEP datasets used in this study

Ref	Compound(s)	Strain	Time points	Stage[a]	Controls	[Drug]	Rep.	GEO no	Date accessed
(Tarr *et al*., 2011)	Thiostrepton~	3D7	24 h	R	DMSO	IC_{50}	3	GSE28701	2019/02
(van der Watt *et al*., 2018)	MMV390048 or MMV642943	3D7	24 or 48 h	R	UT	10 x IC_{50}	1	GSE100692	2019/02
(Gupta *et al*., 2016)	Cisplatin, Etoposide, Methyl ethanesulphonate (MMS), Pyrimethamine	3D7	6 h	R	Reference pool	IC_{50} and IC 90	3	GSE72580	2019/02
(Shaw *et al*., 2015)	dihydroartemisinin (DHA)	K1	1-3 h	T	Not clear	IC_{50}	5	GSE62136	2019/03
(Abd Razak *et al*., 2014)	Choline kinase inhibitor, hexadecyltrimethylam monium bromide	K1	72 h	R	UT	IC_{50}	3	GSE54775	2019/03
(Guler *et al*., 2013)	Novel dihydroorotate dehydrogenase (DHODH) inhibitor	Dd2	Not clear	Not clear	Dd2	IC_{50}	3	GSE35732 GSE37306	2019/03
(Brunner *et al*., 2012)	ACT-213615	3D7	1, 2, 4, 6, and 8 h	T	DMSO	IC_{50}	unclear	GSE39485	2019/03
(Andrews *et al*., 2012)	Trichostatin A (TSA), suberoylanilide hydroxamic acid (SAHA) and 2aminosuberic acid derivative (2-ASA-9)	3D7	2 h	T	DMSO	IC_{90}	2	GSE25642	2019/02
(Kritsiriwuthinan *et al*., 2011)	Pyronaridine, CQ	K1	4 h and 24 h	T	UT	IC_{50}	3	GSE31109 GSE30867 GSE30869	2019/02
(Becker *et al*., 2010)	Cyclohexylamine	3D7	18, 25 and 30 hpi	Both	UT	IC_{99}	2	GSE18075	2019/02
(van Brummelen *et al*., 2008)	DL-α-difluoromethylornithine (DFMO)	3D7	19, 27 and 34 hpi	Both	UT	5x IC_{50}	2	GSE13578	2019/03
(Becker *et al*., 2011)	Dehydrobrachylaenolide	3D7	2, 6, and 12 h	Both	DMSO	IC_{99}	2	GSE29874	2019/03
(Hu *et al*., 2009)	ML7, W7, KN7, Staurosporine, KN93, Cyclosporine A, FK506, Roscovitine A, Quinine, Chloroquine, Febrifugine, artemisinin, Na3VO4, Colchicine, Retinol A, PMSF, E64, Leupeptine, Apicidin, Trichostatin A, EGTA	3D7	1,2,4,6,8 and 10 h	Both	UT	IC_{50} and IC_{90}	1 or 2	GSE19468	2018/06
(Cheemadan *et al*., 2014)	Ionomycin	3D7	30 min, 1, 2, 4 and 6 h	S	Reference pool	10x IC_{50}	1	GSE33869	2019/02

[a]: R= rings, T = trophozoites; S = schizonts; UT= untreated parasites, hpi = hours post invasion, h= hours, min= minutes , Rep. = replicates

Table S2: Filtering criteria imposed on GEP datasets

**Criteria**	**Accepted**	**Rejected**
Controls	Untreated parasites under same conditions as compound-treated parasites	Different conditions compared to compound-treated
Gene coverage	>65% coverage of *P. falciparum* genes	<65% coverage of *P. falciparum* genes
Mode of action	Known in *P. falciparum*	Unknown in *P. falciparum*
Time series	If there are ≥2 time points available for comparison to other compound treatments	Compound treatments that have no time points or replicates
Concentrations	IC_{50} and higher concentrations	Concentrations below IC_{50}
Parasite strain	Treatments and controls need to be the same strain, preferably NF54 or 3D7	Resistant strains or clinical isolates will not be considered as transcriptional responses may vary due to strain differences and not compound treatments

Table S3: Final database generated from 6 datasets spanning 20 compound treatments

Compound	Mode of action (MoA)	Ref for MoA	Dataset	GEO No	Total treatment time points (tp) used	Gene coverage after pre-processing
W7	Calcium/calmodulin-dependent protein kinase inhibitor	(Hu *et al.*, 2009;Brunner *et al.*, 2012)	(Hu *et al.*, 2009)	GSE19468	160/248 dataset tp<br><br>W7= all exp7 tp<br><br>ML-7= all exp8 tp<br><br>Staurosporine= all exp14 tp<br><br>Cyclosporin A= all exp2 tp<br><br>Colchicine= all exp5 tp<br><br>PMSF= all exp16 tp<br><br>Leupeptin= all exp24 tp<br><br>Artemisinin= all exp11 tp<br><br>Chloroquine= all exp25 tp<br><br>Febrifugine= all exp10 tp<br><br>Quinine= all exp9 tp	3705/5400 (69%)
ML-7		(Hu *et al.*, 2009;Coronado *et al.*, 2016)				
Staurosporine	Inhibits serine/threonine kinases, reduces merozoite invasion	(Dluzewski and Garcia, 1996;Karaman *et al.*, 2008)				
Cyclosporin A	Has a strong affinity to sphingomyelin in membrane environment like parasitized erythrocytes membranes, thus aids in inhibiting merozoite invasion. Also believed to be a calcineurin pathway inhibitor.	(Hu *et al.*, 2009;Dynarowicz-Łątka *et al.*, 2015)				
Colchicine	Microtubule is the target, inhibits merozoite invasion	(Fowler *et al.*, 1998)				
PMSF	Serine protease inhibitor	(Tan-No *et al.*, 2008)				
Leupeptin	A cysteine, serine, and threonine peptidase inhibitor which affects haemoglobin degradation	(Moura *et al.*, 2009)				
Artemisinin	Partially understood but hypothesized to be involved in producing carbon-centered free radicals that in turn alkylate heme and proteins	(Meshnick, 2002)				
Chloroquine	Inhibits the heme polymerase enzyme	(Slater, 1993)				
Febrifugine	Targets *P. falciparum* prolyl-tRNA synthetase activity	(Keller *et al.*, 2012)				
Quinine	Partially understood but accumulate in the parasite's digestive vacuole (DV) and may inhibit the detoxification of heme	(Petersen *et al.*, 2011)				
DFMO	Inhibits ornithine decarboxylase causing parasite arrest	(Assaraf *et al.*, 1987)	(van Brummelen *et al.*, 2008)	GSE13578	3/3 time points with replicates	4050/5400 (75%)
MMV 048 and MMV 943	Inhibits *Plasmodium* phosphatidylinositol 4kinase (PI4K)	(Brunschwig *et al.*, 2018)	(van der Watt *et al.*, 2018)	GSE100692	6/10 dataset tp<br>MMV 048= all asexual tp<br>MMV 943= all asexual tp	4971/5400 (92%)
ACT-213615	Artemisinin derivative that has an unknown MoA which is different from other antimalarials based different transcriptional responses to that of the Hu *et al.* dataset	(Brunner *et al.*, 2012)	(Brunner *et al.*, 2012)	GSE39485	5/5 dataset tp	4857/5400 (90%)
Ionomycin	Increases cytoplasmic calcium concentrations	(Cheemadan *et al.*, 2014)	(Cheemadan *et al.*, 2014)	GSE33869	5/10 dataset tp<br>Ionomycin= all schizont tp	4495 /5400 (83%)
Trichostatin A (TSA), Suberoylanilide hydroxamic acid, 2aminosuberic acid derivative, Apicidin	Histone deacetylase (HDAC) inhibitors that perturb the transcriptome	(Darkin-Rattray *et al.*, 1996;Hu *et al.*, 2009;Andrews *et al.*, 2012)	(Hu *et al.*, 2009)	GSE19468	Trichostatin A = all epx21 tp	3705/5400 (69%)
			(Andrews *et al.*, 2012)	GSE25642	6/6 dataset tp<br>1 tp per treatment, 2 replicates per treatment	4364/5400 (80%)

Table S4: Optimal Hyperparameter tuning ranges for algorithms

Algorithm	Hyperparameter	Tuning range	Interval	Category	R tuning package
Support vector machine<br>Polynomial kernel (P)<br>Sigmoid kernel (S0<br>Linear kernel (L)<br>Radial kernel (R)	Gamma		P= (0, 0.1, 0.3, 0.5, 1, 2, 4, 8, 10)<br><br>S= (0, 0.1, 0.3, 0.5, 1, 2, 4, 8, 10)<br><br>L= (0, 0.1, 0.3, 0.5, 1, 2, 4, 8, 10)<br><br>R= (0.5,1,2)		e1071
	Degrees		P= (1, 2, 3, 4, 5, 6)<br>L= (1, 2, 3, 4, 5, 6)		
	Cost		P=10-3:1010<br>S= 10^{-3}:10^{10}<br>L= 10^{-3}:10^{10}<br>R= 10^{-1}:10^{2}		
Multinomial logistic regression	N/A	-	-	-	-
Random Forest (RandomForest)	Number of trees		1,10,100, 500,1000, 5000		e1071
	Mtries		6, 10, 20		
Random Forest (h2o package)	ntrees		100,250, 500,1000, 5000		h2o
	Mtry		1,5,10,15,20		
	Max depth		2,3,4,5,6		
Gradient boosting machine (h2o)	Number of trees	100-4000	100,200,300, 400,500, 1000,4000	-	h2o,
	col_sample_rate	0.3-1	0.3, 0.7, 1.0	-	
	max_depth	4-20	4,6,8,12,<br>16, 20	-	
Gradient boosting machine (Xgboost)	Col sample rate	0.1:1			caret
	Max depth		2, 3, 4, 5, 6		
	Subsample	0.1:1			
	nrounds		50, 100, 150		
	Eta		0.025, 0.05, 0.1, 0.3		
Artificial neural network	Activation function	-	-	Rectifier,<br>RectifierWithDropout<br>Maxout,<br>MaxoutWithDropout	h2o
	Hidden drop out ratio	0-0.3	(0,0), (0.15,0.15), (0.3,0.3)	-	
	Input drop out ratio	0-0.3	0, 0.15, 0.3	-	
	L1 and L2 regularization	0-0.1	0,0.00001, 0.0001, 0.001, 0.01, 0.1	-	
	Adaptive rate	0.005-0.02	0.005, 0.01, 0.015, 0.02	-	
	Loss function	-	-	Automatic,<br>CrossEntropy,<br>Quadratic, Huber,<br>Absolute, Quantile	

Table S5: Optimal hyperparameters identified from hyperparameter tuning

Algorithm	Hyperparameter	Optimal Hyperparameter for biomarkers	Optimal Hyperparameter for database	Logloss	Classification error	Out-of-bag error	Accuracy	R tuning package
Support vector machine<br>• Polynomial kernel (P)<br>• Sigmoid kernel (S)<br>• Linear kernel (L)<br>• Radial kernel (R)	Gamma	P=0.1<br>S=0.1<br>L=0<br>R=0	P=0.1<br>S=0.1<br>L=0<br>R=0	N/A	B:<br>P= 0.14<br>S= 0.5<br>L= 0.15<br>R= 0.8<br><br>D:<br>P= 0.25<br>S= 0.7<br>L= 0.17<br>R= 0.8	N/A	N/A	e1071
	Degrees	P=1<br>L=1	P=1<br>L=1					
	Cost	P=1<br>S=1000<br>L=0.1<br>R=0.001	P=0.1<br>S=0.1<br>L=0.01<br>R=0.001					
Multinomial logistic regression	N/A	N/A	N/A	N/A	N/A	N/A	N/A	N/A
RandomForest	Number of trees	460	4000	N/A	B= 0.26<br>D= 0.29	N/A	N/A	e1071, RandomForest
	Mtries	6	6	N/A	N/A	B= 29.27%<br>D= 23.17%	N/A	
Random Forest (h2o)	ntrees	500	1000	B= 0.957<br>D= 0.995	N/A	N/A	N/A	h2o
	Mtry	6	6					
	Max depth	20	20					
Xgboost	Col sample rate	0.6	0.6	N/A	N/A	N/A	B= 78.87%<br>D= 77.24%	caret
	Max depth	1	2					
	Subsample	0.75	0.75					
	Nrounds	50	50					
	Min child weight	1	1					
	Eta	0.4	0.4					
Gradient Boosting Machine	col_sample_rate	0.3	0.3	B= 2.30x 10^{-8}<br>D= 1.15x 10^{-6}	N/A	N/A	N/A	h2o
	max_depth	6	4					
	Ntrees	500	100					
Artificial neural network	Activation function	MaxoutWithDropout	MaxoutWithDropout	B= 0.001<br>D= 0.001	N/A	N/A	N/A	h2o
	Hidden drop out ratio	0.15	0.3					
	Input drop out ratio	0.3	0.3					
	L1 regularization	1.0x 10^{-5}	0.01					
	L2 regularization	0	0.001					
	Adaptive rate	false	false					
	Loss function	Automatic	Automatic					

Note: D= database model, B= biomarker model

Table S6: The gene ID of the 174 genes extracted using our rational gene selection

Row	Gene IDs								
1	PF3D7_1112700 unknown	PF3D7_1249400 unknown	PF3D7_0814300 AAA family ATPase, putative	PF3D7_0511800 inositol-3-phosphate synthase	PF3D7_1308500 unknown	PF3D7_0718500 prefoldin subunit 3, putative	PF3D7_0730900 EMP1-trafficking protein	PF3D7_1214100 GPI ethanolamine phosphate transferase 3, putative	PF3D7_1306200 unknown
2	PF3D7_1307800 FHA domain-containing protein, putative	PF3D7_0813200 CS domain protein, putative	PF3D7_0527900 ATP-dependent RNA helicase DDX41, putative	PF3D7_1337300 exoribonuclease, putative	PF3D7_1230900 serine/threonine protein kinase RIO1, putative	PF3D7_1462300 GTP-binding protein, putative	PF3D7_0220300 Plasmodium exported protein, unknown function	PF3D7_0405700 lysine decarboxylase, putative	PF3D7_0614300 major facilitator superfamily-related transporter, putative
3	PF3D7_0919700 pyridoxal phosphate homeostasis protein, putative	PF3D7_1352000 GTP-binding protein, putative	PF3D7_0806600 kinesin-like protein, putative	PF3D7_0416200 unknown	PF3D7_0206100 cysteine desulfuration protein SufE	PF3D7_0310500 ATP-dependent RNA helicase DHX57, putative	PF3D7_1127900 unknown	PF3D7_1317100 DNA replication licensing factor MCM4	PF3D7_0503400 actin-depolymerizing factor 1
4	PF3D7_0103600 ATP-dependent DNA/RNA helicase PSH1	PF3D7_1444100 unknown	PF3D7_0814400 phospholipase DDHD1, putative	PF3D7_1324000 unknown	PF3D7_0815200 importin subunit beta, putative	PF3D7_1220400 debranching enzyme-associated ribonuclease, putative	PF3D7_0309600 60S acidic ribosomal protein P2	PF3D7_0505300 UDP-N-acetylglucosamine transporter, putative	PF3D7_0802100 AP2 domain transcription factor, putative
5	PF3D7_1422400 nucleolar RNA-associated protein, putative	PF3D7_0504200 ATP-dependent RNA helicase DDX27, putative	PF3D7_1364300 pre-mRNA-splicing factor ATP-dependent RNA helicase PRP16	PF3D7_1331700 glutamine--tRNA ligase, putative	PF3D7_0626400 CRAL/TRIO domain-containing protein, putative	PF3D7_1217900 PPPDE peptidase domain-containing protein, putative	PF3D7_1031300 SAE2 domain-containing protein, putative	PF3D7_1430700 NADP-specific glutamate dehydrogenase	PF3D7_1463800 ribosomal protein S6, mitochondrial, putative
6	PF3D7_1458900 golgi apparatus membrane protein TVP23, putative	PF3D7_0624000 hexokinase	PF3D7_0321800 WD repeat-containing protein, putative	PF3D7_1133800 RNA (uracil-5-) methyltransferase, putative	PF3D7_1218300 AP-2 complex subunit mu	PF3D7_1124100 BEACH domain-containing protein, putative	PF3D7_0509100 structural maintenance of chromosomes protein 4, putative	PF3D7_1476200 Plasmodium exported protein (PHISTb), unknown function	PF3D7_0612600 cytoplasmic tRNA 2-thiolation protein 1, putative
7	PF3D7_1223600 unknown	PF3D7_1475100 unknown	PF3D7_1434600 methionine aminopeptidase 2	PF3D7_1336000 unknown	PF3D7_1438000 eukaryotic translation initiation factor eIF2A, putative	PF3D7_0508700 pre-mRNA-processing ATP-dependent RNA helicase PRP5, putative	PF3D7_1142600 60S ribosomal protein L35ae, putative	PF3D7_0411000 unknown	PF3D7_0704500 serine/threonine protein kinase, putative
8	PF3D7_0929000 transcription initiation factor TFIID subunit 7, putative	PF3D7_0317300 unknown	PF3D7_1251700 tryptophan--tRNA ligase	PF3D7_0108700 secreted ookinete protein, putative	PF3D7_1024900 unknown	PF3D7_1019800 tRNA methyltransferase, putative	PF3D7_1107400 DNA repair protein RAD51	PF3D7_0301800 Plasmodium exported protein, unknown function	PF3D7_0618100 unknown
9	PF3D7_1039000 serine/threonine protein kinase, FIKK family	PF3D7_1115400 cysteine proteinase falcipain 3	PF3D7_0924100 unknown	PF3D7_1235500 N6-adenosine-methyltransferase, putative	PF3D7_0603100 RNA-binding protein, putative	PF3D7_1304900 DNA-directed RNA polymerase II subunit RPB11, putative	PF3D7_1211700 DNA replication licensing factor MCM5, putative	PF3D7_1016800 Plasmodium exported protein (PHISTc), unknown function	PF3D7_1038400 gametocyte-specific protein
10	PF3D7_0514900 unknown	PF3D7_0516300 tRNA pseudouridine synthase, putative	PF3D7_0308900 splicing factor 3B subunit 1, putative	PF3D7_0623900 ribonuclease H2 subunit A, putative	PF3D7_1407400 unknown	PF3D7_1142300 conserved Plasmodium membrane protein, unknown function	PF3D7_0220100 DnaJ protein, putative	PF3D7_1340900 sodium-dependent phosphate transporter	PF3D7_0612200 leucine-rich repeat protein
11	PF3D7_0220000 liver stage antigen 3	PF3D7_1245900 ankyrin-repeat protein, putative	PF3D7_1427000 unknown	PF3D7_0525200 structural maintenance of chromosomes protein 6, putative	PF3D7_0302000 pre-mRNA-splicing factor PRP46, putative	PF3D7_1474500 splicing factor 3A subunit 1, putative	PF3D7_1452400 unknown	PF3D7_0213000 unknown	PF3D7_0604100 AP2 domain transcription factor
12	PF3D7_1364000 unknown	PF3D7_0717800 unknown	PF3D7_1107700 pescadillo homolog	PF3D7_1030600 tRNA N6-adenosine threonylcarbamoyl transferase	PF3D7_1437000 N-acetyltransferase, GNAT family, putative	PF3D7_1439300 Sad1/UNC domain-containing protein, putative	PF3D7_1411400 plastid replication-repair enzyme	PF3D7_1252400 reticulocyte binding protein homologue 3, pseudogene	PF3D7_1325400 CRWN-like protein, putative
13	PF3D7_1120700 unknown	PF3D7_1013500 phosphoinositide-specific phospholipase C	PF3D7_1467400 50S ribosomal protein L22, apicoplast, putative	PF3D7_0808100 AP-3 complex subunit delta, putative	PF3D7_0208800 protein P22, putative	PF3D7_0711000 AAA family ATPase, CDC48 subfamily	PF3D7_1011400 proteasome subunit beta type-5	PF3D7_1008700 tubulin beta chain	PF3D7_0713500 unknown

14	PF3D7_0823800 DnaJ protein, putative	PF3D7_1322200 TOG domain-containing protein, putative	PF3D7_1324700 SNARE protein, putative	PF3D7_1342000 40S ribosomal protein S6	PF3D7_0917500 unknown	PF3D7_1015500 Nucleotidyl transferase, putative	PF3D7_1242700 40S ribosomal protein S17, putative	PF3D7_1340300 nucleolar complex protein 2, putative	PF3D7_1316200 ADP-ribosylation factor, putative
15	PF3D7_1440500 allantoicase, putative	PF3D7_0110000 unknown	PF3D7_1106800 pseudo-tyrosine kinase-like protein	PF3D7_0206700 adenylosuccinate lyase	PF3D7_1234800 splicing factor 3B subunit 3, putative	PF3D7_0914500 unknown	PF3D7_1425800 unknown	PF3D7_1367100 U1 small nuclear ribonucleoprotein 70 kDa homolog, putative	
16	PF3D7_0810500 protein phosphatase PPM7, putative	PF3D7_0619800 conserved Plasmodium membrane protein, unknown function	PF3D7_0526900 transmembrane emp24 domain-containing protein, putative	PF3D7_1447900 multidrug resistance protein 2	PF3D7_0628700 unknown	PF3D7_0719600 60S ribosomal protein L11a, putative	PF3D7_0609900 unknown	PF3D7_0715500 ATP synthase subunit epsilon, mitochondrial, putative	
17	PF3D7_1414000 26S proteasome regulatory subunit RPN13, putative	PF3D7_0709900 hydrolase, putative	PF3D7_0811200 ER membrane protein complex subunit 1, putative	PF3D7_1219000 formin 2	PF3D7_1132000 ubiquitin-like protein, putative	PF3D7_1008800 nucleolar protein 5, putative	PF3D7_1001600 exported lipase 2	PF3D7_0203700 protein MAK16, putative	
18	PF3D7_1323800 vacuolar protein sorting-associated protein 52, putative	PF3D7_1359000 unknown	PF3D7_0910300 unknown	PF3D7_0106700 small ribosomal subunit assembling AARP2 protein	PF3D7_1414200 unknown	PF3D7_1212700 eukaryotic translation initiation factor 3 subunit A, putative	PF3D7_0409600 replication protein A1, large subunit	PF3D7_0525700 unknown	
19	PF3D7_0305100 unknown	PF3D7_1457900 unknown	PF3D7_0322100 mRNA-capping enzyme subunit beta	PF3D7_1244100 N-alpha-acetyltransferase 15, NatA auxiliary subunit, putative	PF3D7_0606600 unknown	PF3D7_1226300 haloacid dehalogenase-like hydrolase, putative	PF3D7_1351000 phosphatidylinositol transfer protein, putative	PFI0905w*	
20	PF3D7_1456700 unknown	PF3D7_0827100 translation initiation factor IF-2, putative	PF3D7_1366600 signal recognition particle receptor subunit alpha, putative	PF3D7_0614800 endonuclease III-like protein 1, putative	PF3D7_1013900 translation initiation factor eIF-2B subunit delta, putative	PF3D7_1315700 tRNA (adenine(58)-N(1))-methyltransferase catalytic subunit TRM61, putative	PF3D7_1124900 60S ribosomal protein L35, putative	PF3D7_1404400 ribosomal protein L16, mitochondrial, putative	

*New gene ID could not be found, unknown= conserved *Plasmodium* protein, unknown function

Table S7: Overlap between 50 biomarker genes and Cowell *et al.* druggable genome.

**Treatment where biomarker was identified**	**Gene ID**	**Gene Product**	**Drugs used to produce compound resistant clones** (Cowell and Winzeler, 2018;Cowell *et al.*, 2018)
Biomarker genes that were found to have copy number variants (CNVs) in compound-resistant clones in Cowell *et al.* study			
ML-7 & W7	PF3D7_0108700	Secreted ookinete protein [a]	MMV019662, MMV028038, MMV665882, GNF179
Trichostatin A	PF3D7_0322100	mRNA-capping enzyme subunit beta	MMV006767
	PF3D7_1039000	Serine/threonine protein kinase, FIKK family	MMV026596
	PF3D7_1112700	Conserved *Plasmodium* protein, unknown function	BRD1095
Staurosporine A	PF3D7_1220400	Debranching enzyme-associated ribonuclease, [a]	MMV665852
DFMO	PF3D7_0509100	Structural maintenance of chromosomes protein 4, [a]	MMV673482
Cyclosporine A	PF3D7_0317300	Conserved Plasmodium protein, unknown function	MMV006767
Ionomycin	PF3D7_1038400	Gametocyte-specific protein	MMV019066, MMV026596
MMV'048 & UCT'943	PF3D7_0301800	*Plasmodium* exported protein, unknown function	MMV665924
Biomarker genes that were found to have single nucleotide variants (SNVs) and insertions or deletions (indels) discovered in compound-resistant clones			
Staurosporine A	PF3D7_1220400	Debranching enzyme-associated ribonuclease, [a]	MMV006767
	PF3D7_1317100	DNA replication licensing factor MCM4	Atovaquone
DFMO	PF3D7_0503400	Actin-depolymerizing factor 1	Atovaquone
Cyclosporine A	PF3D7_1352000	GTP-binding protein, [a]	MMV006767, MMV007224
Chloroquine & Quinine	PF3D7_1322200	Conserved *Plasmodium* protein, unknown function	Cladosporin
PMSF	PF3D7_0823800	DnaJ protein, [a]	Atovaquone
	PF3D7_1115400	Cysteine proteinase falcipain 3	Atovaquone
MMV'048 & UCT'943	PF3D7_1340900	Sodium-dependent phosphate transporter	Atovaquone

[a] = putative

1.2 Supplementary Figures

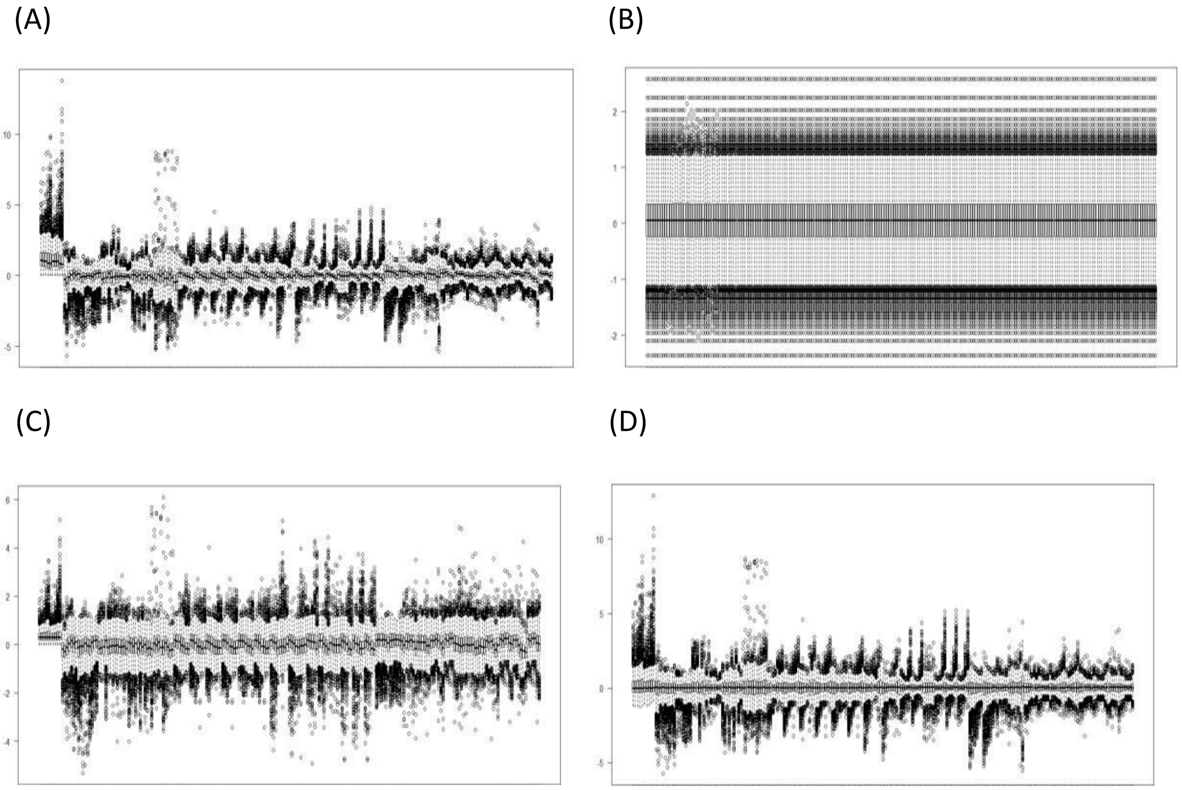

Supplementary Figure S1. Normalization strategies applied to the 2463-gene database. (A) Unnormalized vs different array normalization strategies were implemented such as (B) quantile normalization, (C) medium scaling normalization and (D) cyclic loess normalization. Data from 6 datasets (Supplementary Table S3) was used, with a total of 200 time points (i.e., treatment and control time points), ranged over 20 different compound treatments.

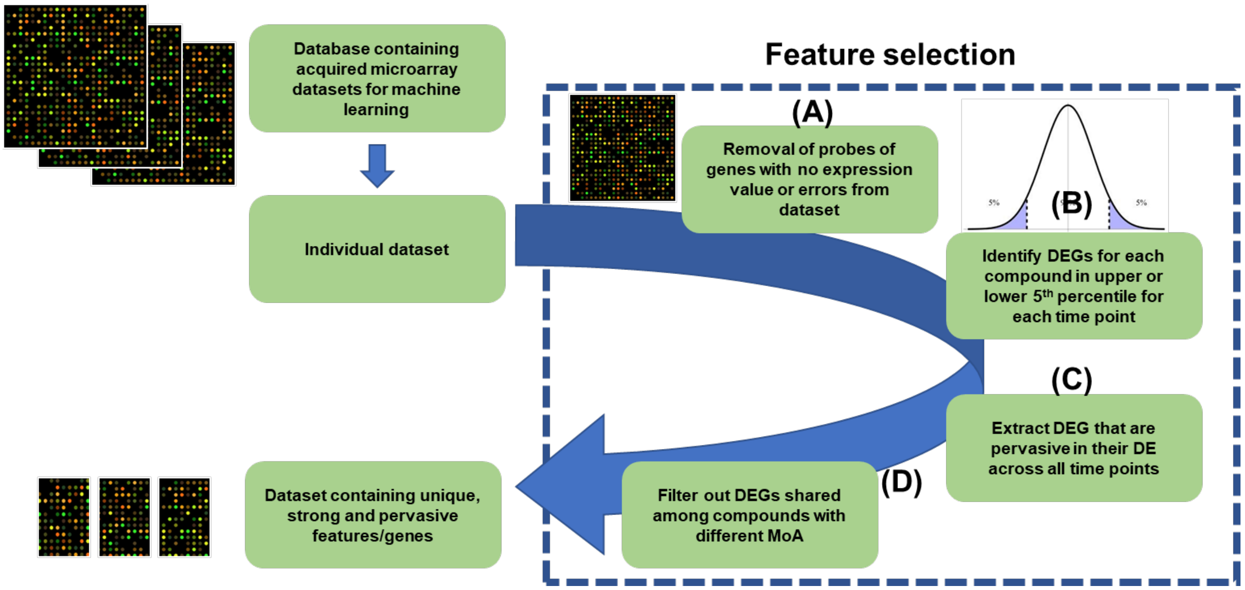

Supplementary Figure S2. Feature selection filtering process to identify biomarker genes with unique predictive features. From our database containing the accepted GEP datasets of compound-treated *P. falciparum*, individual datasets undergo feature selection whereby biomarker genes, i.e., important features for predictive modelling are identified. (A) Each dataset is pre-processed to remove gene probes with no signal. (B) After which DEGs are identified for each treatment and all their corresponding time points. (C) DEGs identified for each compound are filtered to extract DEGs that are pervasively DE across all time points for that compound. (D) The extracted DEGs with pervasive DE are then filtered to exclude DEGs shared among compounds with different MoA.

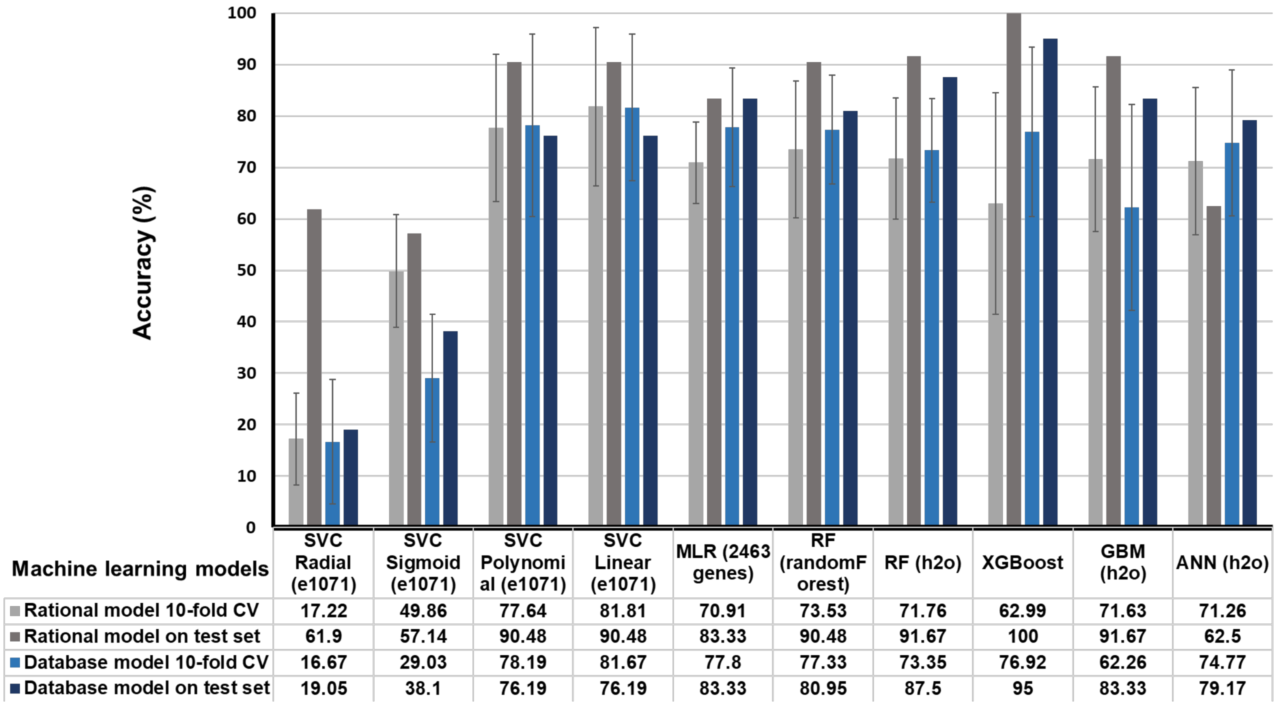

Machine learning models	SVC Radial (e1071)	SVC Sigmoid (e1071)	SVC Polynomial (e1071)	SVC Linear (e1071)	MLR (2463 genes)	RF (randomForest)	RF (h2o)	XGBoost	GBM (h2o)	ANN (h2o)
Rational model 10-fold CV	17.22	49.86	77.64	81.81	70.91	73.53	71.76	62.99	71.63	71.26
Rational model on test set	61.9	57.14	90.48	90.48	83.33	90.48	91.67	100	91.67	62.5
Database model 10-fold CV	16.67	29.03	78.19	81.67	77.8	77.33	73.35	76.92	62.26	74.77
Database model on test set	19.05	38.1	76.19	76.19	83.33	80.95	87.5	95	83.33	79.17

Supplementary Figure S3. Investigated algorithms' model performance comparison. Algorithms classifiers were either trained on the 2463-gene inclusive database (blue) or 175-gene rational selected database (gray). Classifiers were hyperparameter tuned before undergoing 10-fold cross-validation. Bars indicate the accuracy the classifier obtained from either the 10-fold cross-validation (light color) on the training data or accuracy in stratifying the MoA of test data (dark color). SVC= support vector classification, RF=random forest, GBM=gradient boosting machine, ANN= artificial neural network. R packages are shown in brackets.

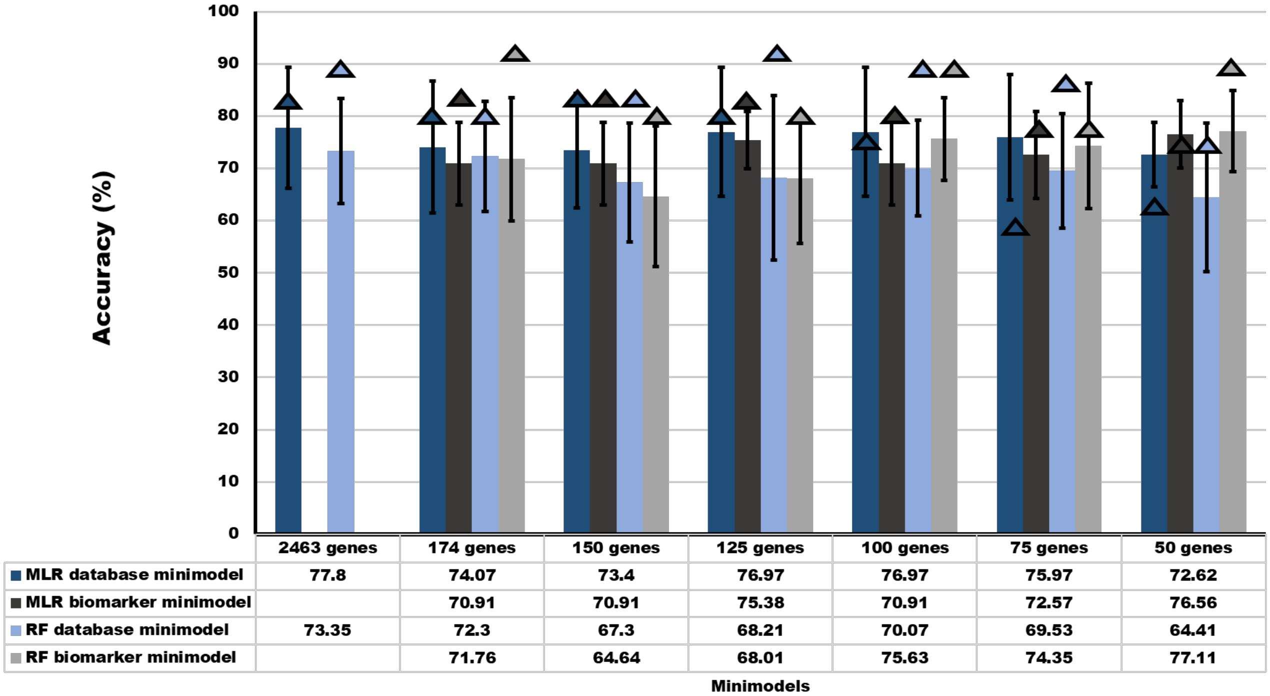

	2463 genes	174 genes	150 genes	125 genes	100 genes	75 genes	50 genes
MLR database minimodel	77.8	74.07	73.4	76.97	76.97	75.97	72.62
MLR biomarker minimodel		70.91	70.91	75.38	70.91	72.57	76.56
RF database minimodel	73.35	72.3	67.3	68.21	70.07	69.53	64.41
RF biomarker minimodel		71.76	64.64	68.01	75.63	74.35	77.11

Minimodels

Supplementary Figure S4. Influence of limiting the number of genes used for training on MoA stratification of MLR and RF models. MLR and RF classifiers were trained on either ML-inferred features (dark and light blue respectively) or on rationally selected features (dark and light gray respectively). Using variable importance, genes were ranked according to their importance in making classification decisions for the classifier. With the ranked genes a sliding gene-scale approach was applied where the top genes were used to make minimodels with each sequential model containing decreasing number of genes/features used to train the classifier. Minimodels underwent 10-fold cross-validation and was also assessed in the accuracy of MoA stratification on test data.

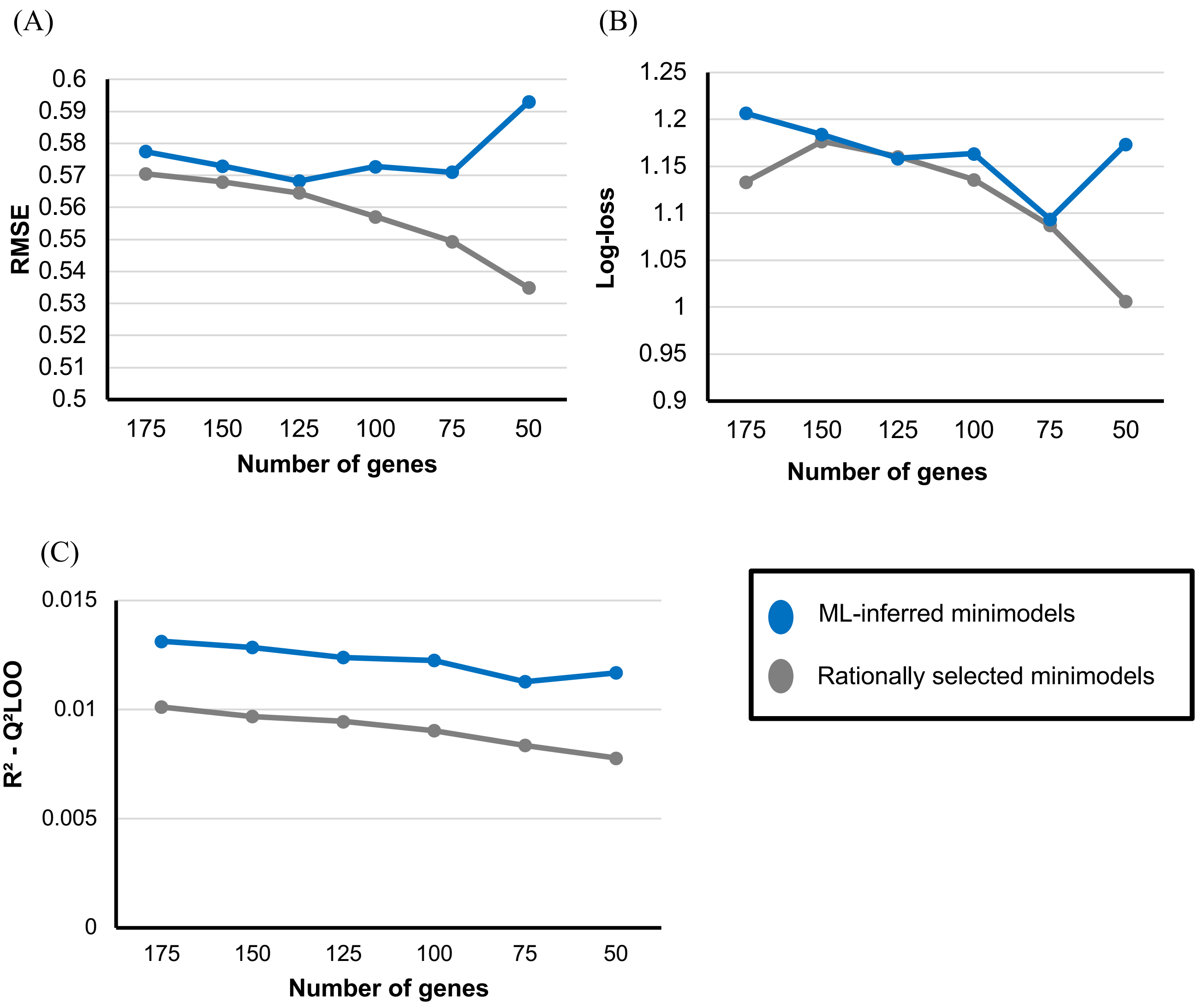

Supplementary Figure S5. Influence of limiting the number of genes used for training on RMSE, log-loss and Q^2LOO of MLR minimodels. MLR classifiers were trained on either ML-inferred genes (dark blue) or on rationally selected genes (dark gray). Using variable importance, genes were ranked according to their importance in making classification decisions for the classifier. With the ranked genes a sliding gene-scale approach was applied where the top genes were used to make minimodels with each sequential model containing decreasing number of genes/features used to train the classifier. The respective minimodels underwent leave-one-out cross validation (LOOCV), whereby the root mean squared error (RMSE), average log-loss and the LOOCV correlation coefficient (Q^2LOO) was calculated. (A) The RMSE calculated during LOOCV for both minimodels trained on the ML-inferred and those trained on the rationally selected genes. This trend becomes stops for ML-inferred minimodels once fewer than 125 genes are used. (B) The average log-loss calculated during LOOCV for the respective minimodels. (C) The difference between the R^2 and Q^2LOO for minimodels using the ML-inferred and rationally selected genes.

2 References

1. Tarr SJ, Nisbet RER, Howe CJ. Transcript-level responses of Plasmodium falciparum to thiostrepton. Molecular and biochemical parasitology. 2011;179(1):37-41.

2. van der Watt ME, Reader J, Churchyard A, Nondaba SH, Lauterbach SB, Niemand J, *et al.* Potent Plasmodium falciparum gametocytocidal compounds identified by exploring the kinase inhibitor chemical space for dual active antimalarials. Journal of Antimicrobial Chemotherapy. 2018;73(5):1279-90.

3. Gupta DK, Patra AT, Zhu L, Gupta AP, Bozdech Z. DNA damage regulation and its role in drug-related phenotypes in the malaria parasites. Scientific reports. 2016;6(1):1-15.

4. Shaw PJ, Chaotheing S, Kaewprommal P, Piriyapongsa J, Wongsombat C, Suwannakitti N, *et al.* Plasmodium parasites mount an arrest response to dihydroartemisinin, as revealed by whole transcriptome shotgun sequencing (RNA-seq) and microarray study. BMC genomics. 2015;16(1):1-14.

5. Abd Razak MRM, Abdullah NR, Chomel R, Muhamad R, Ismail Z. Effect of choline kinase inhibitor hexadecyltrimethylammonium bromide on Plasmodium falciparum gene expression. Southeast Asian Journal of Tropical Medicine and Public Health. 2014;45(2):259.

6. Guler JL, Freeman DL, Ahyong V, Patrapuvich R, White J, Gujjar R, *et al.* Asexual populations of the human malaria parasite, Plasmodium falciparum, use a two-step genomic strategy to acquire accurate, beneficial DNA amplifications. PLoS Pathog. 2013;9(5):e1003375.

7. Brunner R, Aissaoui H, Boss C, Bozdech Z, Brun R, Corminboeuf O, *et al.* Identification of a new chemical class of antimalarials. The Journal of infectious diseases. 2012;206(5):735-43.

8. Andrews KT, Gupta AP, Tran TN, Fairlie DP, Gobert GN, Bozdech Z. Comparative gene expression profiling of P. falciparum malaria parasites exposed to three different histone deacetylase inhibitors. PloS one. 2012;7(2):e31847.

9. Kritsiriwuthinan K, Chaotheing S, Shaw PJ, Wongsombat C, Chavalitshewinkoon-Petmitr P, Kamchonwongpaisan S. Global gene expression profiling of Plasmodium falciparum in response to the anti-malarial drug pyronaridine. Malaria journal. 2011;10(1):1-10.

10. Becker JV, Mtwisha L, Crampton BG, Stoychev S, van Brummelen AC, Reeksting S, *et al.* Plasmodium falciparum spermidine synthase inhibition results in unique perturbation-specific effects observed on transcript, protein and metabolite levels. BMC genomics. 2010;11(1):1-16.

11. van Brummelen AC, Olszewski KL, Wilinski D, Llinas M, Louw AI, Birkholtz LM. Co-inhibition of Plasmodium falciparum S-adenosylmethionine decarboxylase/ornithine decarboxylase reveals perturbation-specific compensatory mechanisms by transcriptome, proteome, and metabolome analyses. J Biol Chem. 2008;284(7):4635-46.

12. Becker JV, Van der Merwe MM, van Brummelen AC, Pillay P, Crampton BG, Mmutlane EM, *et al.* In vitro anti-plasmodial activity of Dicoma anomala subsp. gerrardii (Asteraceae): identification of its main active constituent, structure-activity relationship studies and gene expression profiling. Malaria journal. 2011;10(1):1-11.

13. Hu G, Cabrera A, Kono M, Mok S, Chaal BK, Haase S, *et al.* Transcriptional profiling of growth perturbations of the human malaria parasite Plasmodium falciparum. Nature Biotechnology. 2009;28:91.

14. Cheemadan S, Ramadoss R, Bozdech Z. Role of calcium signaling in the transcriptional regulation of the apicoplast genome of Plasmodium falciparum. BioMed research international. 2014;2014.

15. Coronado LM, Montealegre S, Chaverra Z, Mojica L, Espinosa C, Almanza A, *et al*. Blood Stage Plasmodium falciparum Exhibits Biological Responses to Direct Current Electric Fields. PloS one. 2016;11(8):e0161207.

16. Karaman MW, Herrgard S, Treiber DK, Gallant P, Atteridge CE, Campbell BT, *et al*. A quantitative analysis of kinase inhibitor selectivity. Nature biotechnology. 2008;26(1):127-32.

17. Dluzewski A, Garcia C. Inhibition of invasion and intraerythrocytic development of Plasmodium falciparum by kinase inhibitors. Experientia. 1996;52(6):621-3.

18. Dynarowicz-Łątka P, Wnętrzak A, Makyła-Juzak K. Cyclosporin A in membrane lipids environment: implications for antimalarial activity of the drug—the Langmuir monolayer studies. The Journal of membrane biology. 2015;248(6):1021-32.

19. Fowler R, Fookes R, Lavin F, Bannister L, Mitchell G. Microtubules in Plasmodium falciparum merozoites and their importance for invasion of erythrocytes. Parasitology. 1998;117(5):425-33.

20. Tan-No K, Shimoda M, Sugawara M, Nakagawasai O, Niijima F, Watanabe H, *et al*. Cysteine protease inhibitors suppress the development of tolerance to morphine antinociception. Neuropeptides. 2008;42(3):239-44.

21. Moura PA, Dame JB, Fidock DA. Role of Plasmodium falciparum digestive vacuole plasmepsins in the specificity and antimalarial mode of action of cysteine and aspartic protease inhibitors. Antimicrobial agents and chemotherapy. 2009;53(12):4968-78.

22. Meshnick SR. Artemisinin: mechanisms of action, resistance and toxicity. International journal for parasitology. 2002;32(13):1655-60.

23. Slater AF. Chloroquine: mechanism of drug action and resistance in Plasmodium falciparum. Pharmacol Ther. 1993;57(2-3):203-35.

24. Keller TL, Zocco D, Sundrud MS, Hendrick M, Edenius M, Yum J, *et al*. Halofuginone and other febrifugine derivatives inhibit prolyl-tRNA synthetase. Nature chemical biology. 2012;8(3):311.

25. Petersen I, Eastman R, Lanzer M. Drug-resistant malaria: molecular mechanisms and implications for public health. FEBS letters. 2011;585(11):1551-62.

26. Assaraf Y, Golenser J, Spira D, Messer G, Bachrach U. Cytostatic effect of DL-α-difluoromethylornithine against Plasmodium falciparum and its reversal by diamines and spermidine. Parasitology research. 1987;73(4):313-8.

27. Brunschwig C, Lawrence N, Taylor D, Abay E, Njoroge M, Basarab GS, *et al*. UCT943, a next-generation Plasmodium falciparum PI4K inhibitor preclinical candidate for the treatment of malaria. Antimicrobial agents and chemotherapy. 2018;62(9).

28. Darkin-Rattray SJ, Gurnett AM, Myers RW, Dulski PM, Crumley TM, Allocco JJ, *et al*. Apicidin: a novel antiprotozoal agent that inhibits parasite histone deacetylase. Proceedings of the National Academy of Sciences. 1996;93(23):13143-7.

Section 2: Machine learning theory

2.1 Hyperparameter testing

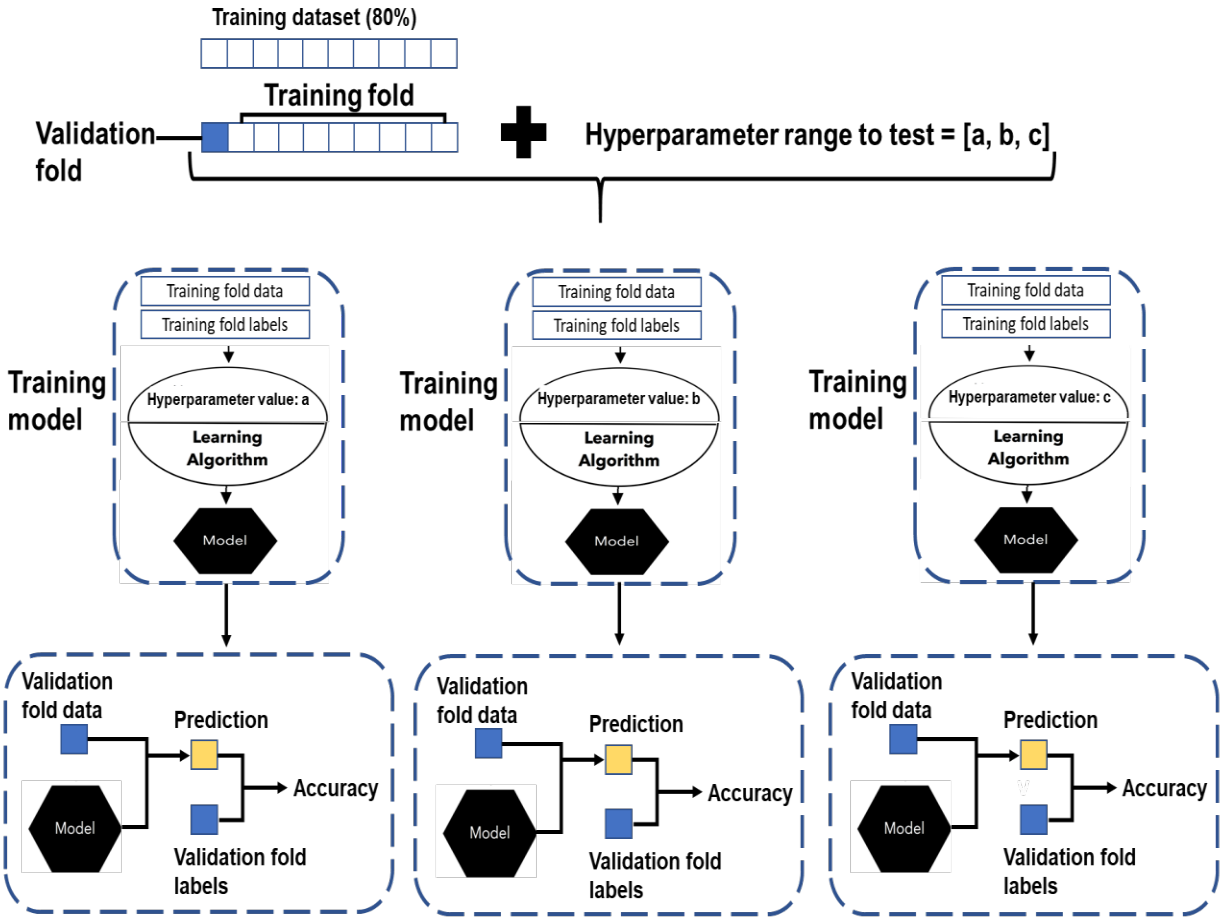

Supplementary Figure S6. Principle of hyperparameter tuning. A hyperparameter range or grid is given to the ML algorithm, whereby the ML algorithm trains on the training fold and builds a model/classifier using a hyperparameter value within the range or grid to define the model's architecture. For each hyperparameter value given a model is built and the performance of the model assessed. This can also be done to assess different combinations of different hyperparameter values. The hyperparameter values which gives the model the best accuracy is then identified.

2.2 Principle of multiclassification support vector machines

In machine learning, SVM is a supervised algorithm and can be separated into two categories, namely Support Vector Regression (SVR) and Support Vector Classification (SVC) (Gholami and Fakhari, 2017). For the purpose of this study which addresses a classification problem, only SVCs

will be considered. SVMs were originally developed to solve binary problems by identifying the optimal separating linear hyperplane that can separate and differentiate between members and non-members of a given class in an abstract space as shown in Supplementary Figure S7 (Brown *et al.*, 1999).

As seen in Supplementary Figure S7, there can be multiple hyperplanes that can separate the two classes, but not all hyperplanes will perform as well in classifying members of the circle class that are situated close to members of the square class. The SVM algorithm thus selects the optimal hyperplane which has the maximum margin i.e., distance from observations of each class (Gholami and Fakhari, 2017).

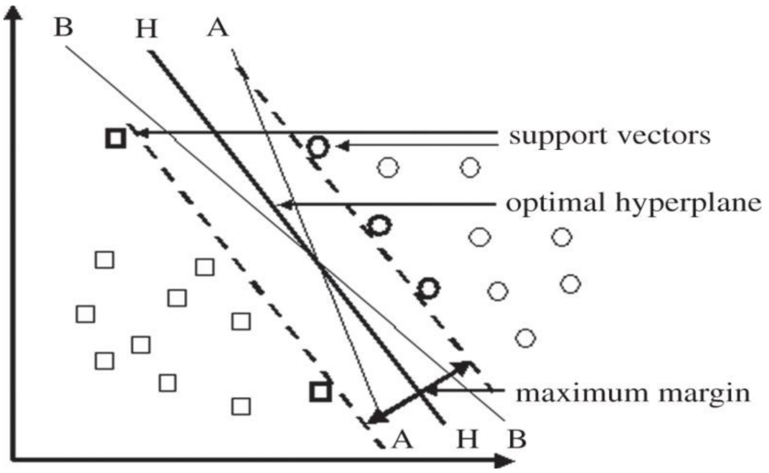

Supplementary Figure S7.: **Principle of SVM classification.** The squares and dots are spread onto a 2D feature space (not restricted to 2D) based on their respective properties. The SVM algorithm then produces multiple hyperplanes (A, H, B) to help separate the two classes (dots and squares). SVM then assesses each hyperplane in their ability to separate the two classes with the maximum distance between the two classes. Source: (Hepworth *et al.*, 2012)

Not all observations, however, are linearly separable, e.g., Supplementary Figure S8, and thus one solution SVM uses is to create a nonlinear feature space by applying a “kernel trick,” whereby the observations of the two classes can then be separated by the hyperplane (Frunza, 2016;Yahyaoui's *et al.*, 2018). This kernel is a statistical mapping function which allows nonlinear data to be transformed into a higher dimension that will allow separation of different classes by a hyperplane (Wittek, 2014).

Although SVC had been developed to address binary classification problems, real-life classification problems are multi-class and thus the algorithm has been adapted to address these problems as well (Frunza, 2016). The ‘one-against-one’ approach is an example such of a method developed by Knerr *et al.* which is used to implement a multi-class SVM, where several classifiers are combined (Knerr *et al.*, 1990).

Each classifier is binary and is built based on its’ training on two of the *n* classes, thereby resulting in *n*(*n*-1)/2 classifiers (Knerr *et al.*, 1990;Frunza, 2016). For new data, each of these classifiers is applied and classification is made for each classifier resulting in a vector of individual classifications

being created, e.g. AAB. From these individual classifications, the final class is identified by majority vote, which in this case is class A.

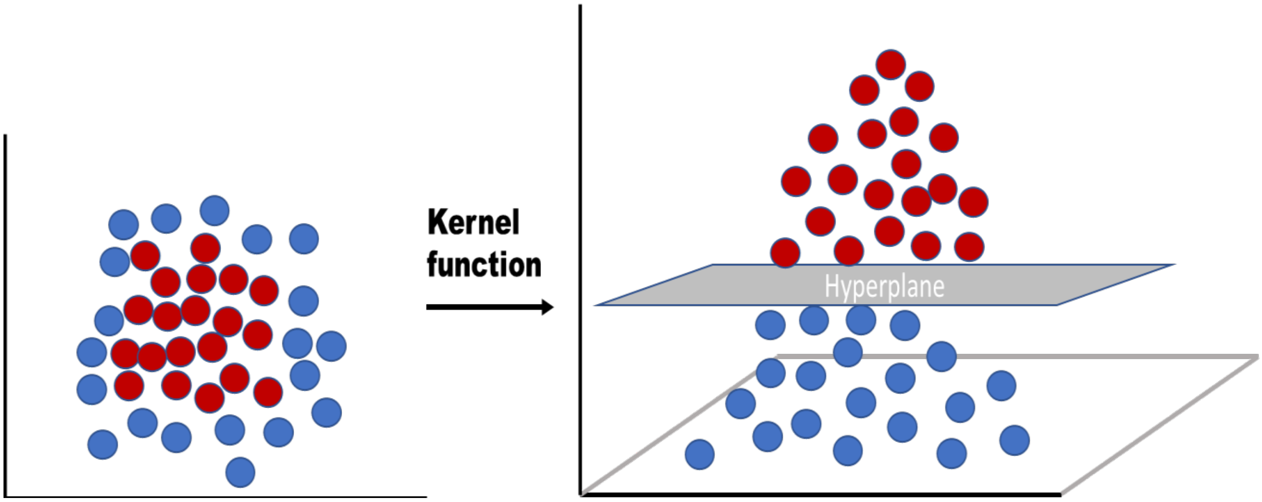

Supplementary Figure S8.: **Support vector machine kernel function to separate nonlinear data.** Support vector machines apply a kernel function to transform the data into a higher-dimensional space whereby the nonlinear data of two groups (red and blue) can be separated with a hyperplane whereas this would not have been accomplished linearly (Jakkula, 2006).

2.3 Principle of multinomial logistic regression

Machine learning extensively uses statistics and mathematic tools to help build a model from the training data it is given so it can predict or classify new data. Logistic regression (LR) is an example of such statistical tools used in ML and is similar to linear regression. With linear regression a linear relationship is assumed between the input variables and the output variable and a generalized linear model (GLM) is built that describes this linear relation (Nylen and Wallisch, 2017). However, in cases where the data is not linearly correlated and/or the output variable is discontinuous or categorical in nature, it is more beneficial to use logistic regression than linear regression (Hoffman, 2019). Since our problem is categorical classification and we cannot assume that the GEP data are linearly correlated, LR is more useful.

The principle behind the LR algorithm is that it uses a sigmoid function to calculate the probability of whether an object belongs to a class or not (Nylen and Wallisch, 2017;Hoffman, 2019). It does this by estimating the coefficients (parameter/beta weights) that link the input variables to the outcome variable using a maximum likelihood estimation approach (Nylen and Wallisch, 2017). Yet few real-world classification problems are binary but rather multi-class, such as ours.

The multinomial logistic regression approach was developed to address such multiclass problems, in which log odds of outcomes (logit values as shown in Supplementary Figure S9) are modelled as a linear combination of the input variables (Fávero and Belfiore, 2019). A logit value is the natural logarithmic probability of an event, such as belonging to a class. However, these values as seen in

Supplementary Figure S9, do not add up to one. Hence a softmax function is used to transform these values into probability distributions of a list of potential classes/ outcomes (Ouyed and Allili, 2018). To help identify the predicted class the cross-entropy function is applied, which measures the distance of these probabilities for each class and the list of classes within the model and selects the one which has the shortest distance.

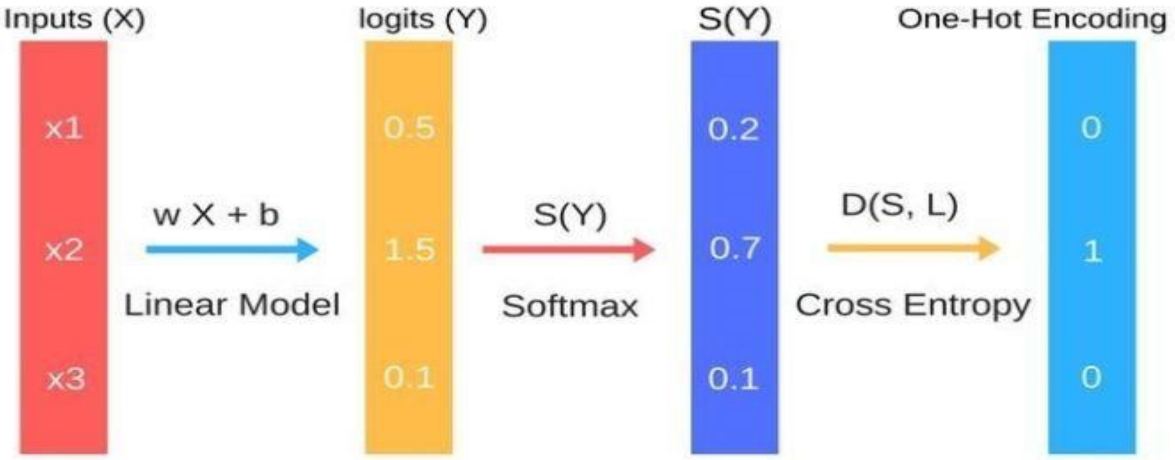

Supplementary Figure S9.: **Multinomial logistic regression algorithm**. The multinomial logistic algorithm analyses each input i.e. feature and builds a linear model for each input so that each input has its own weight (w) which is applied to a feature during the training phase of the algorithm. Each model will produce a logit score that with the help of a softmax function can convert the score into the probability of belonging to a class. Cross entropy calculates the distance between the probabilities for each class and selects the class with the shortest distance as the output. Source:(Polamuri, 2017)

2.4 Principle of random forest

Random forest is an ensemble classifier that employs decision trees and bootstrap aggregating (Genuer *et al*., 2017). Ensemble classifiers is a machine learning technique that combines several base models to build an optimal model with better performance (Fratello and Tagliaferri, 2019). Random forests (RF), for example, create multiple decision trees and the output from these decision trees helps it make a classification as shown in Supplementary Figure S10 and is much more powerful and accurate than a single decision tree (Breiman, 2001). In principle of decision trees, the training dataset is repeatedly partitioned until the data can no longer be split. At the root of the decision tree, which contains the whole dataset, a feature is identified and a decision rule made that will employ a splitting criterion (Fratello and Tagliaferri, 2019).

At this node the data will be partitioned into subsets, wherewith each subset a feature is again selected and a split criterion implemented until the data is no longer able to be split (Breiman, 2001). With RF, multiple trees are made, but the algorithm does not select the data points or variables in each of the decision trees. Rather it randomly samples the data points and variables from each of these trees that it creates and combines the output and makes a vote on the class (Cao *et al*., 2012).

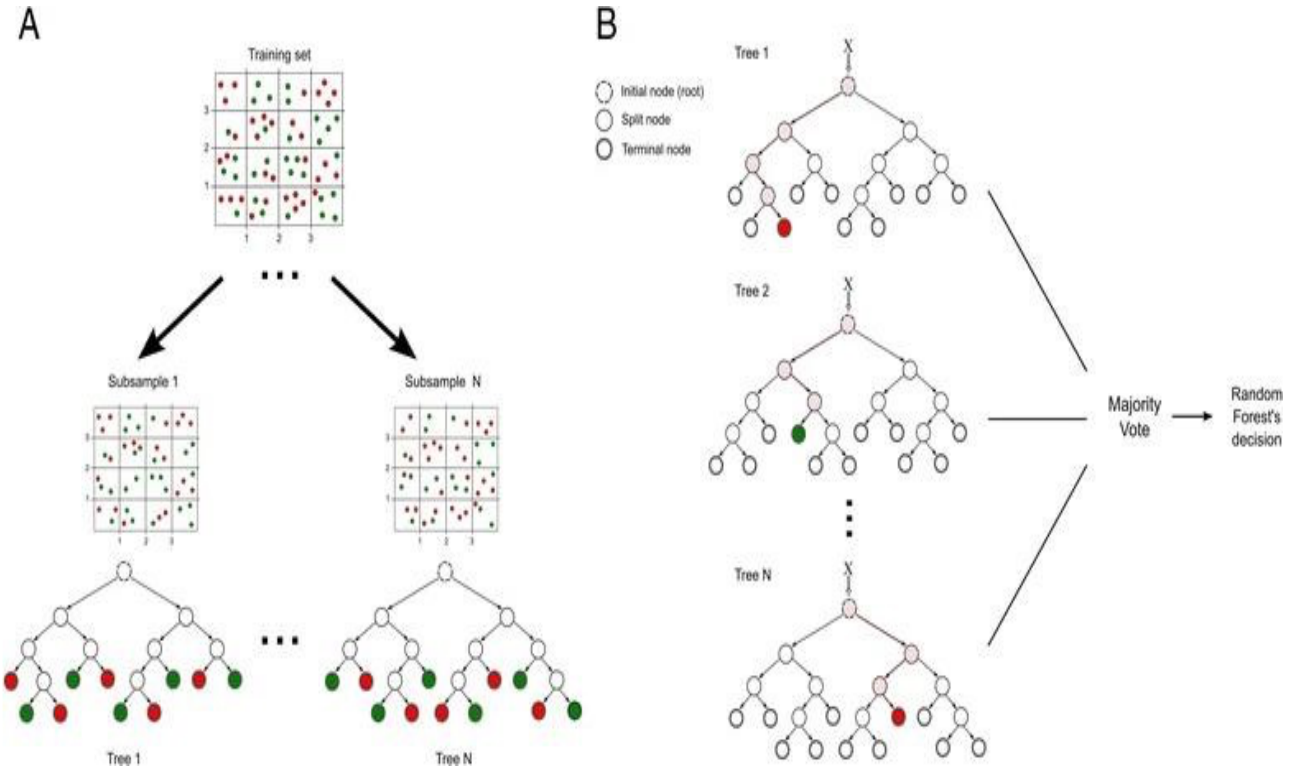

Supplementary Figure S10. Random forest employing bootstrap aggregation and multiple decision trees. A) From the training data, the algorithm applies bootstrap aggregating whereby subsets of the training data are used to build a decision tree. B) To predict the class of new input data, the algorithm takes the decision of all decision trees into account and uses a majority vote to identify the class (green and red), which in this case is the red class. For each new input data (X), the algorithm starts at the root of the tree and based on intrinsic properties of the data selects a branch to transverse down the tree until a leaf is reached whereby the class decision is made. This is done simultaneously for several decision trees. Source:(Machado *et al*., 2015)

2.5 Principle of gradient boosting machines

Another ensemble classifier, called gradient boosting machines (GBM), has gained wide interest in recent years in their ability to efficiently identify patterns for multiclassification problems. GBMs have been successfully applied in face detection, iris recognition, speech and multiclass text categorization (Ferreira and Figueiredo, 2012).

Gradient boosting machines are similar random forest trees in that it also combines several simple base models to obtain a model with better accuracy, but how this is done differs. GBM builds an initial tree-based model and the next consecutive tree-model is built in such a way as to mitigate the faults of the previous tree-model (Golden *et al*., 2019). This self-correction will continue until an additive model which minimizes the error is found, or the number of trees specified is reached (Touzani *et al*., 2018).

2.6 Principle of artificial neural networks

Artificial neural networks (ANN) has gained a lot of popularity in recent years as it has shown a remarkable ability to process information of biological systems that are prone to nonlinearity, noise, high parallelism and their ability to generalize (Basheer and Hajmeer, 2000).

ANN is a deep machine learning approach that is more advanced than the previously stated algorithms, in that it can gradually extract higher-level features from raw data using multilayered processing units (LeCun *et al*., 2015). An ANN in its' simplest form contains an input layer, hidden layer, and output layer as illustrated in Supplementary Figure S11. Within these layers are nodes that can be fully or partially connected to nodes in other layers (Chen *et al*., 2018).

The input layer contains nodes that represent the input variables of the model and these input variables are transformed using an activation function as they pass through to the hidden nodes. As these transformed variables are fed into the output nodes, output values are calculated that help in making a classification or prediction (Shi, 2014). The number of output nodes corresponds to the number of classes or prediction variables. ANNs are powerful in that each node in the hidden layer functions as a processing unit that can consider all the variables or only a subset and analyse the relationships between these variables (Chen *et al*., 2018). Not only this, but ANNs can also add weights to links connecting nodes as well as self-correct themselves during their training phase by using backpropagation. The ANNs do this self-correction by comparing the output values to the actual values and then adjust the weights on connecting links of nodes accordingly and reassesses the error between the output to actual values (Elbayoumi *et al*., 2015). This is done repeatedly until the ANNs predictive and/or classification performance is optimized.

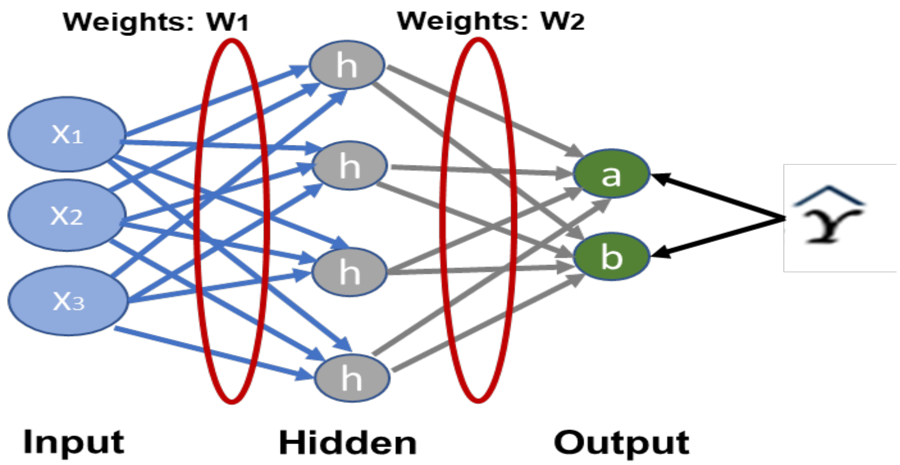

Supplementary Figure S11.: **Simple artificial neural network**. Neural networks have input nodes where data (X) are fed into a hidden layer where hidden nodes can assess information from the input nodes. This hidden layer can be extended to multiple layers and the hidden nodes (processing units) can also be increased. This hidden layer then connects to output nodes which can be increased to the

number of classes or events. The hidden nodes give to each output node/class a probability of being true based on the input information fed into the input layer.

References

Abd Razak, M.R.M., Abdullah, N.R., Chomel, R., Muhamad, R., and Ismail, Z. (2014). Effect of choline kinase inhibitor hexadecyltrimethylammonium bromide on Plasmodium falciparum gene expression. *Southeast Asian Journal of Tropical Medicine and Public Health* 45**,** 259.

Andrews, K.T., Gupta, A.P., Tran, T.N., Fairlie, D.P., Gobert, G.N., and Bozdech, Z. (2012). Comparative gene expression profiling of P. falciparum malaria parasites exposed to three different histone deacetylase inhibitors. *PloS one* 7**,** e31847.

Assaraf, Y., Golenser, J., Spira, D., Messer, G., and Bachrach, U. (1987). Cytostatic effect of DL-α-difluoromethylornithine against Plasmodium falciparum and its reversal by diamines and spermidine. *Parasitology research* 73**,** 313-318.

Basheer, I.A., and Hajmeer, M. (2000). Artificial neural networks: fundamentals, computing, design, and application. *Journal of Microbiological Methods* 43**,** 3-31.

Becker, J.V., Mtwisha, L., Crampton, B.G., Stoychev, S., Van Brummelen, A.C., Reeksting, S., Louw, A.I., Birkholtz, L.-M., and Mancama, D.T. (2010). Plasmodium falciparum spermidine synthase inhibition results in unique perturbation-specific effects observed on transcript, protein and metabolite levels. *BMC genomics* 11**,** 1-16.

Becker, J.V., Van Der Merwe, M.M., Van Brummelen, A.C., Pillay, P., Crampton, B.G., Mmutlane, E.M., Parkinson, C., Van Heerden, F.R., Crouch, N.R., and Smith, P.J. (2011). In vitro anti-plasmodial activity of Dicoma anomala subsp. gerrardii (Asteraceae): identification of its main active constituent, structure-activity relationship studies and gene expression profiling. *Malaria journal* 10**,** 1-11.

Breiman, L. (2001). Random Forests. *Machine Learning* 45**,** 5-32.

Brown, M.P., Grundy, W.N., Lin, D., Cristianini, N., Sugnet, C., Ares, M., and Haussler, D. (1999). Support vector machine classification of microarray gene expression data. *University of California, Santa Cruz, Technical Report UCSC-CRL-99-09*.

Brunner, R., Aissaoui, H., Boss, C., Bozdech, Z., Brun, R., Corminboeuf, O., Delahaye, S., Fischli, C., Heidmann, B., and Kaiser, M. (2012). Identification of a new chemical class of antimalarials. *The Journal of infectious diseases* 206**,** 735-743.

Brunschwig, C., Lawrence, N., Taylor, D., Abay, E., Njoroge, M., Basarab, G.S., Le Manach, C., Paquet, T., Cabrera, D.G., and Nchinda, A.T. (2018). UCT943, a next-generation Plasmodium falciparum PI4K inhibitor preclinical candidate for the treatment of malaria. *Antimicrobial agents and chemotherapy* 62.

Cao, D.-S., Huang, J.-H., Liang, Y.-Z., Xu, Q.-S., and Zhang, L.-X. (2012). Tree-based ensemble methods and their applications in analytical chemistry. *TrAC Trends in Analytical Chemistry* 40**,** 158-167.

Cheemadan, S., Ramadoss, R., and Bozdech, Z. (2014). Role of calcium signaling in the transcriptional regulation of the apicoplast genome of Plasmodium falciparum. *BioMed research international* 2014.

Chen, H., Engkvist, O., Wang, Y., Olivecrona, M., and Blaschke, T. (2018). The rise of deep learning in drug discovery. *Drug Discovery Today* 23, 1241-1250.

Coronado, L.M., Montealegre, S., Chaverra, Z., Mojica, L., Espinosa, C., Almanza, A., Correa, R., Stoute, J.A., Gittens, R.A., and Spadafora, C. (2016). Blood Stage Plasmodium falciparum Exhibits Biological Responses to Direct Current Electric Fields. *PloS one* 11, e0161207.

Cowell, A., and Winzeler, E. (2018). Exploration of the Plasmodium falciparum Resistome and Druggable Genome Reveals New Mechanisms of Drug Resistance and Antimalarial Targets. *Microbiology insights* 11, 1178636118808529-1178636118808529.

Cowell, A.N., Istvan, E.S., Lukens, A.K., Gomez-Lorenzo, M.G., Vanaerschot, M., Sakata-Kato, T., Flannery, E.L., Magistrado, P., Owen, E., Abraham, M., Lamonte, G., Painter, H.J., Williams, R.M., Franco, V., Linares, M., Arriaga, I., Bopp, S., Corey, V.C., Gnädig, N.F., Coburn-Flynn, O., Reimer, C., Gupta, P., Murithi, J.M., Moura, P.A., Fuchs, O., Sasaki, E., Kim, S.W., Teng, C.H., Wang, L.T., Akidil, A., Adjalley, S., Willis, P.A., Siegel, D., Tanaseichuk, O., Zhong, Y., Zhou, Y., Llinás, M., Ottilie, S., Gamo, F.-J., Lee, M.C.S., Goldberg, D.E., Fidock, D.A., Wirth, D.F., and Winzeler, E.A. (2018). Mapping the malaria parasite druggable genome by using in vitro evolution and chemogenomics. *Science* 359, 191-199.

Darkin-Rattray, S.J., Gurnett, A.M., Myers, R.W., Dulski, P.M., Crumley, T.M., Allocco, J.J., Cannova, C., Meinke, P.T., Colletti, S.L., and Bednarek, M.A. (1996). Apicidin: a novel antiprotozoal agent that inhibits parasite histone deacetylase. *Proceedings of the National Academy of Sciences* 93, 13143-13147.

Dluzewski, A., and Garcia, C. (1996). Inhibition of invasion and intraerythrocytic development of Plasmodium falciparum by kinase inhibitors. *Experientia* 52, 621-623.

Dynarowicz-Łątka, P., Wnętrzak, A., and Makyła-Juzak, K. (2015). Cyclosporin A in membrane lipids environment: implications for antimalarial activity of the drug—the Langmuir monolayer studies. *The Journal of membrane biology* 248, 1021-1032.

Elbayoumi, M., Ramli, N.A., and Fitri Md Yusof, N.F. (2015). Development and comparison of regression models and feedforward backpropagation neural network models to predict seasonal indoor PM2.5–10 and PM2.5 concentrations in naturally ventilated schools. *Atmospheric Pollution Research* 6, 1013-1023.

Fávero, L.P., and Belfiore, P. (2019). "Chapter 14 - Binary and Multinomial Logistic Regression Models," in *Data Science for Business and Decision Making,* eds. L.P. Fávero & P. Belfiore. Academic Press), 539-615.

Ferreira, A.J., and Figueiredo, M.a.T. (2012). "Boosting Algorithms: A Review of Methods, Theory, and Applications," in *Ensemble Machine Learning: Methods and Applications,* eds. C. Zhang & Y. Ma. (Boston, MA: Springer US), 35-85.

Fowler, R., Fookes, R., Lavin, F., Bannister, L., and Mitchell, G. (1998). Microtubules in Plasmodium falciparum merozoites and their importance for invasion of erythrocytes. *Parasitology* 117, 425-433.

Fratello, M., and Tagliaferri, R. (2019). "Decision Trees and Random Forests," in *Encyclopedia of Bioinformatics and Computational Biology,* eds. S. Ranganathan, M. Gribskov, K. Nakai & C. Schönbach. (Oxford: Academic Press), 374-383.

Frunza, M.-C. (2016). "Chapter 2I - Support Vector Machines," in *Solving Modern Crime in Financial Markets,* ed. M.-C. Frunza. Academic Press), 205-215.

Genuer, R., Poggi, J.-M., Tuleau-Malot, C., and Villa-Vialaneix, N. (2017). Random Forests for Big Data. *Big Data Research* 9**,** 28-46.

Gholami, R., and Fakhari, N. (2017). "Chapter 27 - Support Vector Machine: Principles, Parameters, and Applications," in *Handbook of Neural Computation,* eds. P. Samui, S. Sekhar & V.E. Balas. Academic Press), 515-535.

Golden, C.E., Rothrock, M.J., and Mishra, A. (2019). Comparison between random forest and gradient boosting machine methods for predicting Listeria spp. prevalence in the environment of pastured poultry farms. *Food Research International* 122**,** 47-55.

Guler, J.L., Freeman, D.L., Ahyong, V., Patrapuvich, R., White, J., Gujjar, R., Phillips, M.A., Derisi, J., and Rathod, P.K. (2013). Asexual populations of the human malaria parasite, Plasmodium falciparum, use a two-step genomic strategy to acquire accurate, beneficial DNA amplifications. *PLoS Pathog* 9**,** e1003375.

Gupta, D.K., Patra, A.T., Zhu, L., Gupta, A.P., and Bozdech, Z. (2016). DNA damage regulation and its role in drug-related phenotypes in the malaria parasites. *Scientific reports* 6**,** 1-15.

Hepworth, P.J., Nefedov, A.V., Muchnik, I.B., and Morgan, K.L. (2012). Broiler chickens can benefit from machine learning: support vector machine analysis of observational epidemiological data. *Journal of The Royal Society Interface* 9**,** 1934.

Hoffman, J.I.E. (2019). "Chapter 33 - Logistic Regression," in *Basic Biostatistics for Medical and Biomedical Practitioners (Second Edition),* ed. J.I.E. Hoffman. Academic Press), 581-589.

Hu, G., Cabrera, A., Kono, M., Mok, S., Chaal, B.K., Haase, S., Engelberg, K., Cheemadan, S., Spielmann, T., Preiser, P.R., Gilberger, T.-W., and Bozdech, Z. (2009). Transcriptional profiling of growth perturbations of the human malaria parasite Plasmodium falciparum. *Nature Biotechnology* 28**,** 91.

Jakkula, V. (2006). Tutorial on support vector machine (svm). *School of EECS, Washington State University* 37.

Karaman, M.W., Herrgard, S., Treiber, D.K., Gallant, P., Atteridge, C.E., Campbell, B.T., Chan, K.W., Ciceri, P., Davis, M.I., and Edeen, P.T. (2008). A quantitative analysis of kinase inhibitor selectivity. *Nature biotechnology* 26**,** 127-132.

Keller, T.L., Zocco, D., Sundrud, M.S., Hendrick, M., Edenius, M., Yum, J., Kim, Y.-J., Lee, H.-K., Cortese, J.F., and Wirth, D.F. (2012). Halofuginone and other febrifugine derivatives inhibit prolyl-tRNA synthetase. *Nature chemical biology* 8**,** 311.

Knerr, S., Personnaz, L., and Dreyfus, G. (Year). "Single-layer learning revisited: a stepwise procedure for building and training a neural network", in: *Neurocomputing*, eds. F.F. Soulié & J. Hérault: Springer Berlin Heidelberg), 41-50.

Kritsiriwuthinan, K., Chaotheing, S., Shaw, P.J., Wongsombat, C., Chavalitshewinkoon-Petmitr, P., and Kamchonwongpaisan, S. (2011). Global gene expression profiling of Plasmodium falciparum in response to the anti-malarial drug pyronaridine. *Malaria journal* 10**,** 1-10.

Lecun, Y., Bengio, Y., and Hinton, G. (2015). Deep learning. *Nature* 521**,** 436.

Machado, G., Recamonde-Mendoza, M., and Corbellini, L. (2015). What variables are important in predicting bovine viral diarrhea virus? A random forest approach. *Veterinary Research* 46**,** 85.

Meshnick, S.R. (2002). Artemisinin: mechanisms of action, resistance and toxicity. *International journal for parasitology* 32**,** 1655-1660.

Moura, P.A., Dame, J.B., and Fidock, D.A. (2009). Role of Plasmodium falciparum digestive vacuole plasmepsins in the specificity and antimalarial mode of action of cysteine and aspartic protease inhibitors. *Antimicrobial agents and chemotherapy* 53**,** 4968-4978.

Nylen, E.L., and Wallisch, P. (2017). "Chapter 7 - Regression," in *Neural Data Science,* eds. E.L. Nylen & P. Wallisch. Academic Press), 189-221.

Ouyed, O., and Allili, M.S. (2018). Feature weighting for multinomial kernel logistic regression and application to action recognition. *Neurocomputing* 275**,** 1752-1768.

Petersen, I., Eastman, R., and Lanzer, M. (2011). Drug-resistant malaria: molecular mechanisms and implications for public health. *FEBS letters* 585**,** 1551-1562.

Polamuri, S. (2017). *How Multinomial Logistic Regression Model Works In Machine Learning* [Online]. @dataaspirant. Available: https://dataaspirant.com/2017/03/14/multinomial-logistic-regression-model-works-machine-learning/ [Accessed 7/19 2019].

Shaw, P.J., Chaotheing, S., Kaewprommal, P., Piriyapongsa, J., Wongsombat, C., Suwannakitti, N., Koonyosying, P., Uthaipibull, C., Yuthavong, Y., and Kamchonwongpaisan, S. (2015). Plasmodium parasites mount an arrest response to dihydroartemisinin, as revealed by whole transcriptome shotgun sequencing (RNA-seq) and microarray study. *BMC genomics* 16**,** 1-14.

Shi, G. (2014). "Chapter 3 - Artificial Neural Networks," in *Data Mining and Knowledge Discovery for Geoscientists,* ed. G. Shi. (Oxford: Elsevier), 54-86.

Slater, A.F. (1993). Chloroquine: mechanism of drug action and resistance in Plasmodium falciparum. *Pharmacol Ther* 57**,** 203-235.

Tan-No, K., Shimoda, M., Sugawara, M., Nakagawasai, O., Niijima, F., Watanabe, H., Furuta, S., Sato, T., Satoh, S., and Arai, Y. (2008). Cysteine protease inhibitors suppress the development of tolerance to morphine antinociception. *Neuropeptides* 42**,** 239-244.

Tarr, S.J., Nisbet, R.E.R., and Howe, C.J. (2011). Transcript-level responses of Plasmodium falciparum to thiostrepton. *Molecular and biochemical parasitology* 179**,** 37-41.

Touzani, S., Granderson, J., and Fernandes, S. (2018). Gradient boosting machine for modeling the energy consumption of commercial buildings. *Energy and Buildings* 158**,** 1533-1543.

Van Brummelen, A.C., Olszewski, K.L., Wilinski, D., Llinas, M., Louw, A.I., and Birkholtz, L.M. (2008). Co-inhibition of Plasmodium falciparum S-adenosylmethionine decarboxylase/ornithine decarboxylase reveals perturbation-specific compensatory mechanisms by transcriptome, proteome, and metabolome analyses. *J Biol Chem* 284**,** 4635-4646.

Van Der Watt, M.E., Reader, J., Churchyard, A., Nondaba, S.H., Lauterbach, S.B., Niemand, J., Abayomi, S., Van Biljon, R.A., Connacher, J.I., Van Wyk, R.D.J., Le Manach, C., Paquet, T., González Cabrera, D., Brunschwig, C., Theron, A., Lozano-Arias, S., Rodrigues, J.F.I.,

Herreros, E., Leroy, D., Duffy, J., Street, L.J., Chibale, K., Mancama, D., Coetzer, T.L., and Birkholtz, L.-M. (2018). Potent Plasmodium falciparum gametocytocidal compounds identified by exploring the kinase inhibitor chemical space for dual active antimalarials. *Journal of Antimicrobial Chemotherapy* 73**,** 1279-1290.

Wittek, P. (2014). "7 - Supervised Learning and Support Vector Machines," in *Quantum Machine Learning,* ed. P. Wittek. (Boston: Academic Press), 73-84.

Yahyaoui's, A., Yahyaoui, I., and Yumuşak, N. (2018). "13 - Machine Learning Techniques for Data Classification," in *Advances in Renewable Energies and Power Technologies,* ed. I. Yahyaoui. Elsevier), 441-450.
